# A prescription‐free, radiobiology‐based framework for automated VMAT planning: A feasibility study in primary prostate cancer radiotherapy

**DOI:** 10.1002/mp.70347

**Published:** 2026-02-26

**Authors:** Dejan Kuhn, Simon K. B. Spohn, Constantinos Zamboglou, Anca‐Ligia Grosu, Dimos Baltas, Ilias Sachpazidis

**Affiliations:** ^1^ Division of Medical Physics Department of Radiation Oncology Medical Center – University of Freiburg Faculty of Medicine University of Freiburg Freiburg im Breisgau Germany; ^2^ Department of Radiation Oncology Medical Center – University of Freiburg Faculty of Medicine University of Freiburg Freiburg im Breisgau Germany; ^3^ Department of Radiation Oncology German Oncology Centre European University Cyprus Limassol Cyprus

**Keywords:** automatic treatment planning, biologically interpreted Pareto analysis, normal tissue complication probability (NTCP), particle swarm optimizaiton (PSO), prescription‐free treatment planning, prostate cancer radiotherapy, radiobiologically guided treatment planning, tumor control probability (TCP), volumetric modulated arc therapy (VMAT)

## Abstract

**Background:**

Current VMAT planning workflows for prostate cancer primarily depend on conventional dose–volume criteria specified at discrete dose or volume points. These point‐based objectives, however, do not necessarily lead to globally optimal, patient‐specific treatment plans. While radiobiological models such as Tumor Control Probability (TCP) and Normal Tissue Complication Probability (NTCP) can provide more meaningful, individualized targets, previous implementations have either employed these for plan evaluation or integrated biological objectives without providing a comprehensive set of deliverable trade‐off plans. To date, no prescription‐free, automated VMAT planning method has been introduced that generates clinically deliverable, patient‐specific Pareto fronts that are biologically interpretable and useful for radiobiological trade‐off analysis.

**Purpose:**

The purpose of this study was to develop and clinically evaluate a fully automated, prescription‐free VMAT planning framework for primary prostate cancer that generates Pareto‐optimal, clinically deliverable treatment plans in radiobiological objective space, constrained by predefined TCP and NTCP levels.

**Methods:**

The proposed framework was implemented within a commercial treatment planning system (TPS). 17 patients with unfavorable intermediate‐risk prostate cancer were retrospectively selected for evaluation. For each patient, TCP and NTCP levels were predefined for three target volumes and seven organs at risk (OARs), restricting the optimization to clinically meaningful regions of the solution space. Plan optimization was performed using Particle Swarm Optimization (PSO) to iteratively adjust VMAT parameters, with the complication‐free tumor control probability (*P_+_*) serving as the sole objective function. All resulting, clinically deliverable plans were generated in the TPS and subsequently analyzed in the bi‐objective radiobiological space defined by injury probability (*P_I_*) versus one minus the benefit probability (1 − *P_B_*). The plan yielding the highest *P_+_* and the corresponding individualized pseudo‐Pareto front were identified for each patient. The proposed method was benchmarked against clinical moderately hypofractionated simultaneous integrated boost (SIB) plans.

**Results:**

The proposed prescription‐independent planning approach successfully generated individualized pseudo‐Pareto fronts for all 17 patients in the radiobiological space of *P_I_* versus (1 − *P_B_*). This enabled clinicians to visualize and interpret trade‐offs between tumor control and normal tissue complication risk within the predefined TCP and NTCP levels. For each patient, the plan with highest *P_+_* achieved superior predicted tumor control and reduced normal tissue toxicity compared to manually optimized clinical plans. The method effectively individualized dose distributions according to patient‐specific anatomy and tumor biology, without reliance on fixed dose prescriptions or conventional constraints. All highest *P_+_* treatment plans fulfilled the clinical dose requirements. Sensitivity analyses demonstrated robustness of the framework with respect to variations in TCP model parameters.

**Conclusion:**

This study demonstrated the feasibility of a fully automated, prescription‐free VMAT planning framework for primary prostate cancer, indicating its potential for future clinical implementation. The proposed framework directly optimized treatment plans in radiobiological objective space, producing Pareto‐optimal, clinically deliverable solutions using predefined TCP and NTCP levels. It enables patient‐specific trade‐off analysis taking into account tumor control and normal tissue complication risk. The work provides a foundation for further development, including the incorporation of geometric uncertainties, acceleration through parallel or GPU‐based computation, and application to additional tumor sites.

## 1 INTRODUCTION

Volumetric Modulated Arc Therapy (VMAT)^1^ represents a major advancement in modern radiotherapy, enabling highly conformal dose delivery through dynamic modulation of the multi‐leaf collimator (MLC) positions, gantry rotation speed, and dose rate during treatment. VMAT is based on inverse optimization principles and allows efficient and precise treatment delivery, even for complex target geometries.

In current clinical practice, VMAT planning is typically guided by rigid dose prescriptions and dose–volume constraints defined for specific volumes of interest (VOIs). These constraints are expressed as discrete points on dose–volume histograms (DVHs) and are converted into objective functions that steer the inverse optimization process. The treatment planning system (TPS) then adjusts the machine parameters according to user‐defined priorities or weights, generating a dose distribution that aims to satisfy the prescribed objectives. However, treatment plans, generated through conventional dose–volume‐based optimization, are not guaranteed to represent globally optimal solutions. Moreover, this approach does not account for radiobiological characteristics of tumor and normal tissues, and the manual, iterative nature of the process makes it time‐consuming and susceptible to inter‐planner variability.^2^

To address these issues, several strategies have been explored to increase the level of automation and consistency in radiotherapy treatment planning (RTP). These include multi‐criteria optimization, knowledge‐based planning approaches (such as atlas‐based or statistical modeling methods) and, more recently, deep learning based techniques.^2^ For instance, Künzel et al.^3^ employed Particle Swarm Optimization (PSO) to refine a template VMAT plan using a discrete plan quality score derived from fixed dose prescriptions.

In parallel, radiobiological response models have been explored as alternatives to purely physical, point‐based dose objectives. These models describe the relationship between radiation dose and biological response, taking into account the entire dose distribution within the tissue rather than isolated point values, thereby enabling a more comprehensive and biologically meaningful assessment. Traditionally, such radiobiological models have been primarily applied for treatment plan evaluation.^4^ Building on this concept, several studies have proposed replacing physical dose objectives in the inverse planning process with biological objective functions, allowing direct optimization of dose distributions according to tissue‐specific dose–response relationships. For example, in intensity‐modulated radiotherapy (IMRT) planning, the Equivalent Uniform Dose (EUD) has been used as a biological objective,^5^, ^6^ Normal Tissue Complication Probability (NTCP) functions have guided organ‐at‐risk (OAR) sparing,^7^ and Tumor Control Probability (TCP) functions have served as target volume objectives.^8^, ^9^

Källman et al.^10^ were among the first to propose replacing conventional prescriptions with a single biological objective function, namely the probability of complication‐free tumor control (*P_+_*), which combines TCP and NTCP models. Building on this theoretical foundation, several studies employed *P_+_* as an optimization objective. For example, Kim and Tomé^11^ applied this concept to IMRT treatment planning for prostate cancer (PCa) using a commercial TPS, and Lu et al.^12^ used *P_+_* for stereotactic body radiotherapy (SBRT) planning in lung cancer. However, both approaches yielded only a single optimized treatment plan, rather than a set of Pareto‐optimal solutions that would allow explicit exploration of trade‐offs between tumor control and normal tissue complication risk.

Some studies have introduced bi–objective pseudo‐Pareto fronts (hereafter referred to as Pareto fronts) to enable direct comparison among radiotherapy plans. Yet these approaches generally include at least one physical dose‐based objective, often tied to target dose constraints, instead of using purely biological metrics such as probability of benefit (*P_B_*) and probability of injury (*P_I_*).^13^, ^14^

Building on these prior efforts and directly addressing their limitations regarding reliance on physical dose metrics, limited biological optimization, and the absence of clinically deliverable Pareto‐optimal solutions, we introduce, to the best of our knowledge, the first fully automated VMAT planning framework that operates exclusively in the biological objective space.

The method enables clinicians to define patient‐specific clinically acceptable TCP and NTCP levels for all relevant VOIs, and automatically generates a Pareto front of clinically deliverable VMAT plans in the bi–objective space defined by *P_I_* versus (1 − *P_B_*). In the proposed framework, PSO iteratively adjusts the objective parameters of a template VMAT plan, systematically exploring the solution space within the predefined TCP/NTCP bounds while optimizing a single biological objective function, *P_+_*. Each candidate solution (represented as a VMAT plan configuration) is generated in a commercial TPS to ensure compliance with machine delivery constraints and to produce a clinically realistic dose distribution. Each resulting plan strictly satisfied the predefined TCP and NTCP levels for each VOI. Among these plans, the one with the highest *P_+_* value is identified as the optimal balance between tumor control and normal tissue sparing.

Beyond identifying a single optimal plan, the proposed framework generates an entire biological Pareto front, providing clinicians with a set of non‐dominated solutions that make trade‐offs between tumor control and toxicity risk explicit and clinically interpretable.

Our methodology requires prior knowledge of radiobiological parameters for the relevant tumor and OAR structures. For this reason, we focused on primary radiotherapy of PCa, where robust and validated radiobiological parameter estimates are available and where our institution has extensive clinical experience.^15^, ^16^, ^17^, ^18^, ^19^, ^20^, ^21^, ^22^, ^23^, ^24^

The primary objectives of this study were to introduce the proposed prescription‐free RTP framework and to assess its feasibility and performance in a clinical planning environment. To this end, the framework was implemented within Eclipse TPS (Version 15.6, Varian Medical Systems, Palo Alto, CA, USA; a Siemens Healthineers company) and applied retrospectively to a cohort of patients previously treated with moderately hypofractionated simultaneous integrated boost (SIB) VMAT for primary PCa. The resulting biological‐optimization‐based plans were compared with conventionally generated clinical plans.

## 2 MATERIALS AND METHODS

### 2.1 Patient and imaging data

In this study, we retrospectively included 17 patients with unfavorable intermediate‐risk PCa who had participated in the HypoFocal–Phase II clinical trial (PSMA–PET‐ and MRI‐based focal dose escalation for primary prostate radiotherapy: a prospective, multicenter, non‐randomized, two‐arm trial, DRKS00017570, ARO 2020‐01) at the Medical Centre–University of Freiburg.^22^, ^23^ According to the trial protocol, each patient had available imaging datasets, comprising a planning‐CT scan, diagnostic multi‐parametric MRI (mpMRI) acquired on a 3T MR scanner, and either a 68Ga PSMA‐ or 18F‐1007‐PSMA–PET/CT scans. All imaging examinations were performed within 3 months prior to treatment initiation.

These imaging modalities were used for precise target volume (TV) and OAR delineation. For the present feasibility study for prescription‐free planning we excluded setup margins, and therefore planning target volumes (PTV), focusing on the gross tumor volumes (GTV) and clinical target volumes (CTV) for planning. A modelling concept for the intrafractional movement and set‐up uncertainties to estimate their influence on the radiobiological predictions for IMRT treatments of PCa has been published by our institution.^21^ Its implementation in the prescription‐free concept is planned for future work.

According to the HypoFocal trial protocol, three TVs were defined (details for imaging and target definition are provided in Spohn et al.,^22^ Zamboglou et al.,^23^ and Spohn et al.^25^). In brief, patients with intermediate‐ to high‐risk localized PCa enrolled in arm A of the trial received moderately hypofractionated RT with focal dose escalation. A total dose of 60 Gy in 20 fractions (3 Gy per fraction) was prescribed to the whole prostate, with a SIB of up to 75 Gy in 20 fractions delivered to the GTV, delineated based on PET and MRI. Based on these, the following volumes were defined for the modelling of the radiobiological response of tumor tissue:

1. *GTV_union_​* that represents the GTV encompassing all tumor regions identified from both the mpMRI and PSMA–PET imaging modalities, defined as their union,
2. *Prostate^−^* is defined as the prostate gland (CTV2 in Hypofocal trial), excluding GTV_union_, and
3. *CTV^−^* is the 3 mm isotropic extracapsular margin around the prostate gland, derived from CTV1 of the Hypofocal trial (prostate + proximal seminal vesicles + 3 mm margin) by excluding the prostate itself.

**FIGURE 1:**
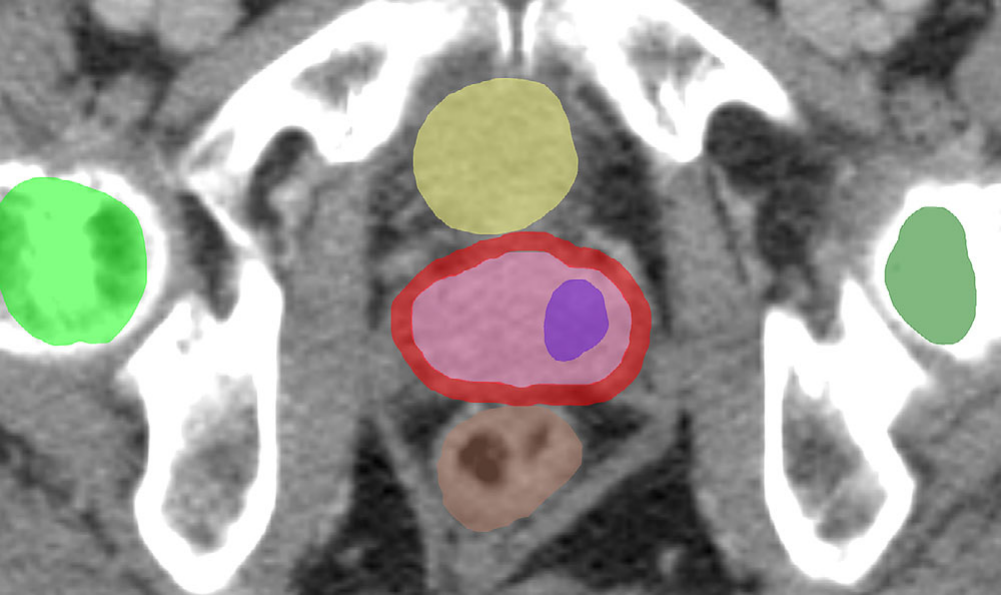
Planning‐CT of an example patient in axial view with TVs and OARs delineated. Visible are GTV_union_ (purple), Prostate^−^ (pink), CTV^−^ (red), Rectum (brown), bladder (yellow), and femoral heads (left: dark green, right: light green).

Additionally, seven OARs were defined: bladder, rectum, small bowel, femoral heads, penile bulb, and sigmoid colon, as illustrated in Figure 1. GTVs were delineated manually by two experienced readers (>4 years) in MRI and PET interpretation. Contouring of OARs was performed according to RTOG guidelines^26^ based on CT imaging, and the CTV was created by following the ESTRO–ACROP guidelines.^27^

In this cohort, the mean prostate gland volume was 41.43 cm^3^ (min: 23.59 cm^3^, max: 60.26 cm^3^). The volume fraction of the GTV_union_ relative to the prostate gland was on average 11.75% (min: 3.67%, max: 39.14%), as shown in Table 1.

**TABLE 1:** Patient‐specific volumes for target structures.

	Volume (cm^3^)				Volume fraction (%)
Case Id	Prostate gland	GTV_union_	Prostate^−^	CTV^−^	GTV_union_/Prostate gland
1	25.64	2.76	22.49	17.23	10.76
2^a^	41.02	6.01	34.76	19.68	14.65
3	60.26	2.21	57.52	30.58	3.67
4	48.67	5.47	42.70	19.51	11.24
5^a^	23.59	1.66	21.85	12.78	7.04
6^a^	55.66	4.62	51.11	25.13	8.30
7	48.01	18.79	28.88	20.69	39.14
8	37.40	3.15	34.15	19.17	8.42
9	35.60	4.75	30.89	19.22	13.34
10	56.73	6.85	49.98	22.46	12.07
11	48.25	3.03	45.28	24.36	6.28
12	42.33	6.98	35.10	21.02	16.49
13	38.41	2.23	36.34	23.25	5.81
14	24.36	3.85	20.63	15.83	15.80
15	38.55	2.32	36.19	17.85	6.02
16	33.16	3.25	29.96	19.74	9.80
17^a^	46.74	5.13	41.61	20.20	10.98
Mean	41.43	4.89	36.44	20.51	11.75
Std	10.73	3.83	10.24	3.87	7.72
Median	41.02	3.85	35.10	19.74	10.76
Min	23.59	1.66	20.63	12.78	3.67
Max	60.26	18.79	57.52	30.58	39.14

Abbreviation: Std, standard deviation.

^a^ Cases chosen for sensitivity analysis were selected based on their proximity to the median and their closeness to the boundaries of the volume fraction.

The dose prescription and the dose–volume constraints defined in the HypoFocal clinical trial are listed in Table 2 for 20 treatment fractions and as iso‐effective dose in 2 Gy fractions (EQD2). These values were not used in the prescription‐free inverse planning process, but they were considered for evaluating the clinical acceptability of the plan solutions that were generated by prescription‐free planning.

**TABLE 2:** Clinical dose constraints for OARs and prescriptions for TVs of the HypoFocal trial.

Structure	Total of 20 fractions	EQD2 (Gy), *α/β *= 1.6 Gy for TVs, *α/β* = 3.0 Gy for OARs
PTV1 (CTV1 + 6 mm margin)	45 Gy in 15 fractions	57.5 Gy
PTV2 (CTV2 + 4 mm margin)	15 Gy in 5 fractions in total 60 Gy in 20 fractions (*D_98%_* ≥ 58.8 Gy, *D_2%_* ≤ 70 Gy)	In total 76.7 Gy (*D_98%_* ≥ 74.2 Gy, *D_2%_* ≤ 99.2 Gy)
PTV3 (GTV_union_ + 2 mm margin)	SIB^a^ near 70 Gy (*D_98%_* ≥ 68.6 Gy, *D_2%_* ≤ 71.4 Gy)	SIB^a^ near 99.2 Gy (*D_98%_* ≥ 95.9 Gy, *D_2%_* ≤ 102.5 Gy)
Rectum	For 60 Gy prescriptions	V_26 Gy_ ≤ 65.4%
	V_20 Gy_ ≤ 85%	V_30 Gy_ ≤ 49.5%
	V_30 Gy_ ≤ 57%	V_40 Gy_ ≤ 38.6%
	V_40 Gy_ ≤ 50%	V_50 Gy_ ≤ 31.5%
	V_50 Gy_ ≤ 35%	V_60 Gy_ ≤ 17.5%
	V_60 Gy_ ≤ 3%	V_70 Gy_ ≤ 2.3%
Bladder	V_40 Gy_ ≤ 50%	V_40 Gy_ ≤ 50%
	V_50 Gy_ ≤ 35%	V_55 Gy_ ≤ 35%
	V_60 Gy_ ≤ 5%	V_72 Gy_ ≤ 5%
	V_67 Gy_ ≤ 1 cm^3^	V_85.1 Gy_ ≤ 1 cm^3^
Femoral head left and right	V_41 Gy_ ≤ 50%	V_41.4 Gy_ ≤ 50%
Small bowel	V_41 Gy_ ≤ 17 cm^3^	V_41.4 Gy_ ≤ 17 cm^3^
	D_max_ ≤ 47 Gy	D_max_ ≤ 50.3 Gy
Sigmoid Colon	V_53 Gy_ ≤ 3 cm^3^	V_59.9 Gy_ ≤ 3 cm^3^
Penile Bulb	V_41 Gy_ ≤ 50%	V_41.4 Gy_ ≤ 50%
	V_49 Gy_ ≤ 20%	V_53.41 Gy_ ≤ 20%

^a^ A simultaneous integrated boost (SIB) of up to 75 Gy was planned; the mean delivered dose was 70 Gy.

### 2.2 Radiobiological models

In the following, we assume a voxel‐based 3D‐anatomy representation and 3D‐dose calculation. To model the biological response of tumor to radiation, we employed the Poisson‐based TCP model.^28^ The Poisson‐based TCP model is defined as follows:

(1) $$TCP=\prod_{i=1}^{M}e^{-\rho \cdot v_{i}\;e^{-\alpha \cdot EQD0_{i}}}$$

with

(2) $$EQD0_{i}=D_{i}\cdot \left(1+\frac{D_{i}/N}{\alpha /\beta }\right)$$

for a total dose D delivered in N fractions, with ρ the clonogenic tumor cell density in cells per cm^3^. Here, we assume a homogeneous cell density across all voxels. $v_{i}$ denotes the volume of the $i^{\mathrm{th}}$ voxel. The parameter α (Gy^−1^) represents the linear sensitivity of clonogenic cells while α/β (Gy) characterizes the fractionation sensitivity according to the Linear–Quadratic (LQ) model.^29^ Finally, *M* is the total number of voxels within the tumor.

To predict the NTCP for OARs, the relative seriality (RS) model^30^ was utilized:

(3) $$NTCP={\left[1-\prod_{i=1}^{M}{\left(1-P{\left(D_{i}\right)}^{s}\right)}^{\frac{v_{i}}{v_{total}}}\right]}^{1/s}$$

where the ratio $v_{i}/v_{total}$ defines the volume fraction being irradiated to dose *D_i_*. The parameter *s* characterizes the degree of seriality of the organ, ranging from values close to zero for parallel structures to higher values for increasingly serial organs. The survival probability for the $i^{th}$ voxel, denoted as $P(D_{i})$, is determined by Equation (4). *M* is similar to Equation (1), the total number of voxels within the organ.

(4) $$P\left(D_{i}\right)=\exp\left(-e^{e\gamma -\left(\frac{EQD2_{i}}{D_{50}}\right)\cdot \left(e\gamma -\ln\ln2\right)}\right)$$

With

(5) $$EQD2_{i}=\frac{EQD0_{i}}{1+\frac{2}{\alpha /\beta }},$$

where *EQD2_i_* is the iso‐effective dose in 2 Gy fractions, *D_50_* is the dose corresponding to a 50% complication probability in EQD2 (Gy), and *γ* is the slope of the response curve.

*P_B_* is defined as the overall TCP, which is achieved across all relevant TVs. In our case, it represents the combined tumor control probability over the different TVs as given by following equation:

(6) $$P_{B}=\prod_{i\in \left\{GTV_{union},Prostate^{-},CTV^{-}\right\}}TCP_{i}$$

Furthermore, *P_I_* for all OARs is calculated as follows:

(7) $$P_{I}=1-\prod_{i=1}^{N_{\mathrm{OARs}}}\left(1-\mathrm{NTC}P_{i}\right),$$

where *N_OARs_* denotes the total number of considered OARs. Finally, *P_+_* ^22^ is given by:

(8) $$P_{+}=P_{B}\cdot \left(1-P_{I}\right)$$

The biologically iso‐effective uniform dose $\bar{\bar{D}}$ is defined as the EQD2 (Gy) that, when delivered uniformly, yields the same dose–response, TCP($\vec{D}$) or NTCP($\vec{D}$), as a given non‐uniform dose distribution $\vec{D}$.^31^ Based on the above equations for TCP (Equation 1) and NTCP (Equations 3 and 4) $\bar{\bar{D}}$ for a TV is given by:

(9) $$\bar{\bar{D}}=\frac{\ln\left(\rho \cdot V\right)-\ln\left(-\ln(TCP\left(\vec{D}\right)\right)}{\alpha \cdot \left(1+\left(\frac{2}{\alpha /\beta }\right)\right)}$$

and for an OAR by:

(10) $$\bar{\bar{D}}=\frac{e\gamma -\ln\left(-\ln\left(NTCP\left(\vec{D}\right)\right)\right)}{e\gamma -\ln\ln2}\cdot D_{50}$$

TCP($\vec{D}$) and NTCP($\vec{D}$) are the tumor control and normal tissue complication probability for the given dose distribution $\vec{D}$. The detailed derivation of the formulas can be found in the Supporting Information.

Alternatively to the above presented voxel‐based calculations, Equations (1)–(5), differential dose–volume histograms (dDVH) for the corresponding VOIs can be used. In this case $v_{i}$ is the volume of the $i^{\mathrm{th}}$ bin and *M* is the total number of bins in the dDVH.

### 2.3 Parameter values for the TCP and NTCP models

We considered homogeneous biological parameters within the three TVs, similar to Lühr et al.^32^ The TCP parameter values for the GTV_union_ were according to Vogelius et al.^33^ *α/β* = 1.6 Gy, and according to Spohn et al.^22^ *α = *0.1205 Gy^−1^, and *ρ* = 2.8·10^8^ cells/cm^3^. In the absence of differentiated data, we assume that the cancer cells in all three TVs exhibit the same radiosensitivity as described by the LQ parameters *α* and *α/β*.

To estimate the cell densities for the other two TVs, Prostate^−^ and CTV^−^, we utilized clinical outcome data from the FLAME randomized Phase III trial,^34^ which reported biochemical failure rates up to 7 years as a function of the dose to the GTV. Doses were converted to EQD2 using an *α/β* ratio of 1.6 Gy. Based on these data, we estimated that the HypoFocal regimen of 60 Gy with a SIB of mean 70 Gy in 20 fractions corresponds to a TCP of approximately 99%. The cell densities for Prostate^−^ and CTV^−^ were then fitted such that *P_B_*, calculated across all three TVs using the HypoFocal dose distributions of the 17 patients (Table 1), matched the expected *P_B_* of 99%. The detailed derivation of the expected *P_B_* can be found in the Supporting Information. We further validated the fitted parameter $\rho_{\mathrm{Prostat}e^{-}}$ using the histopathology data of the prospectively enrolled patients in Freiburg published by Zamboglou et al.^35^ In this study the characteristics of intraprostatic satellite lesions histopathologically justified but missed by visual ^68^Ga–PSMA PET/CT interpretation were investigated. Satellite lesions were defined as microscopic tumor foci that were spatially distinct from the primary lesion and so small that they could not be visually identified on the PET/CT images. We calculated the volume ratio of these satellites to the remaining prostate tissue (prostate–main lesion), multiplied it by the assumed uniform cell density of 2.8·10^8^ cells/cm^3^ in the primary lesion,^22^ and thereby estimated an average clonogenic cell density in the remaining prostate (Prostate^−^) of 1.35·10^6^ cells/cm^3^ with a 95% CI of [2.29·10^5^, 2.74·10^6^].

For the NTCP model, we utilized parameter sets for the RS model obtained from the literature,^36^, ^37^ as shown in Table 3.

**TABLE 3:** Parameter values for the relative seriality NTCP model.

Structure	*D_50_* (Gy)	*γ*	*α/β* (Gy)	*s*
Bladder^37^	80	2.59	3.0	1.30
Rectum^37^	80	1.79	3.0	0.75
Sigmoid colon^36^	80	2.20	3.0	0.70
Small Bowel^36^	60	2.10	3.0	0.14
Penile Bulb^36^	70	2.50	3.0	0.70
Femoral Heads^36^	65	2.70	3.0	1.00

### 2.4 Prescription‐free radiobiology‐based radiation treatment planning

In contrast to the common inverse planning process for the VMAT technique, the proposed method of prescription‐free RTP eliminates the need for predefined dose prescriptions by optimizing for the highest *P_+_*. Instead of rigid physical dose constraints, our method determines individualized dose distributions and prescriptions for each patient based on TCP and NTCP models.

A PSO^38^ algorithm governed the overall optimization process for plan generation. Without having direct access to the VMAT optimization function in the Eclipse VMAT optimizer, the entire VMAT optimization process is encapsulated within a PSO framework. This approach uses PSO to drive the Eclipse VMAT optimizer. The goal of the PSO objective function is to maximize *P_+_*, defined as $\max_{x}P_{+}\left(x\right)$, where x represents the feasible set of VMAT optimizer variables *x* = {dose values} ∪ {priorities}.

The prescription‐free RTP framework has been implemented within the Eclipse TPS v15.6, a commercial TPS by Varian Medical Systems, Inc. (Palo Alto, USA) and follows the steps outlined below:

1. **Baseline plan, and initialization**: A baseline VMAT plan is created, serving as a template plan for Eclipse VMAT plan optimization. Additionally, the PSO algorithm is instantiated by creating plan candidates through variation in both the values of the optimization objectives and their priorities.
2. **VMAT plan optimization**: Eclipse TPS optimizes the dose distribution for each plan candidate. Upon completion of the VMAT optimization, the DVHs for each candidate plan are extracted and made available for further evaluation.
3. **Plan evaluation**: Each plan is assessed by computing radiobiological metrics, including TCP (Equation 1), NTCP (Equation 3), *P_B_* (Equation 6), *P_I_* (Equation 7), and *P_+_* (Equation 8).
4. **PSO update**: The PSO algorithm iteratively updates the values of the optimization objectives and priority parameters for all candidate plans towards *P_+_* maximization. This optimization cycle is repeated until a predefined number of iterations is achieved.
5. **Pareto analysis**: Pareto analysis is performed after each iteration to identify the subset of plans that are not dominated by any other plan regarding *P_B_* and *P_I_*, and thus to update the Pareto optimal set, and to generate the Pareto front in the two‐dimensional objective space *P_I_* versus (1 − *P_B_*).
6. **Plan selection**: From the Pareto optimal set, the treatment plan with the highest *P_+_* is selected as the best trade‐off solution, representing the optimal balance between tumor control and normal‐tissue sparing for the individual patient.

The baseline VMAT plan consisted of two, nearly full rotation arcs (clockwise 181°–179° and counterclockwise 179°–181°), with collimator angles of 5° and 355°, respectively. This template plan was designed for the 20 treatment fractions of the HypoFocal trial, and the jaws were fitted to the CTV^−^ with a 5 mm margin.

**FIGURE 2:**
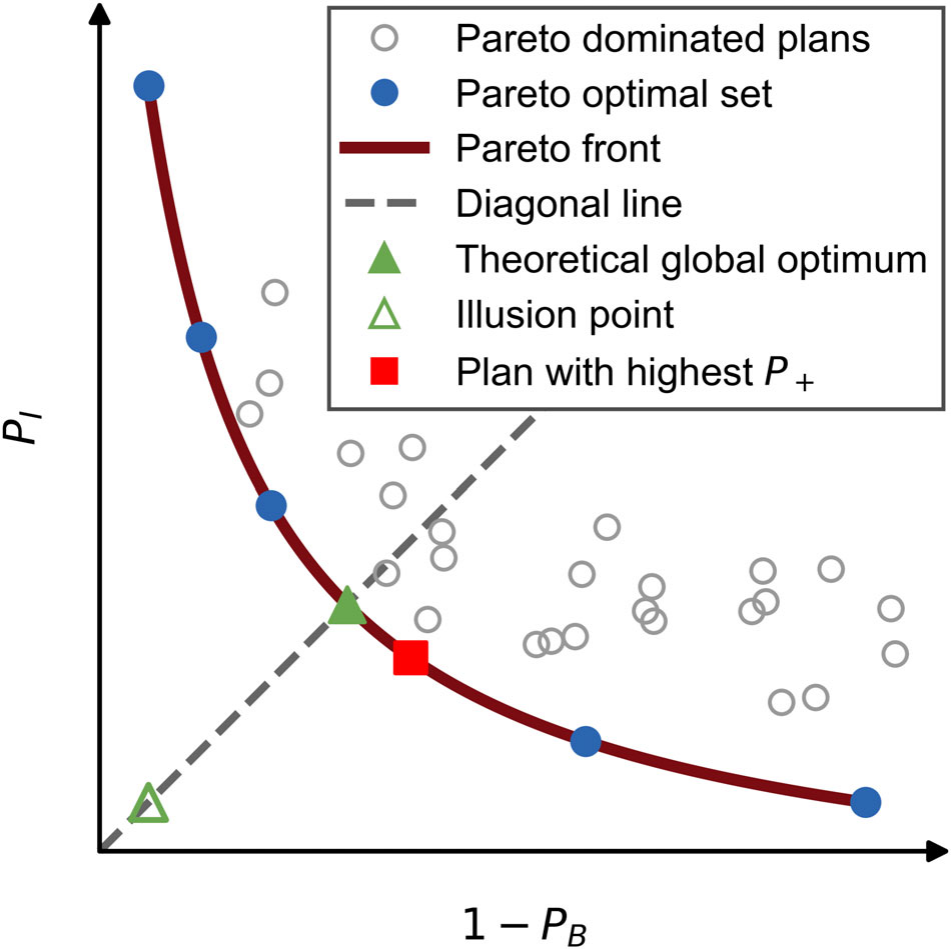
Graphical representation of a Pareto analysis for the prescription‐free treatment planning optimization.

For the VMAT optimizer 13 generalized Equivalent Uniform Dose (gEUD) optimization objectives^39^ were utilized. Specifically, an upper and a lower objective were defined for each TV (GTV_union_, Prostate^−^, and CTV^−^), while a single upper objective was defined for each OAR. Each gEUD objective consists of a gEUD goal value, the volume–effect parameter *a* and an associated priority value. The volume–effect parameter *a* for the gEUD objectives was set to 40 for upper limits and −40 for lower limits, reflecting the permissible boundaries within the TPS. For all 13 gEUD optimization objectives, both the gEUD goal values and the associated priority values were adjusted by PSO. Priority values were sampled over a range from 0 to 1000, corresponding to the allowable priority range of the VMAT optimizer. For the three TVs, the six lower and upper gEUD goal values were sampled within patient‐specific ranges derived from predefined clinically acceptable TCP levels, defined by minimum acceptable and maximum aimed TCP values (see Figure 2).

As shown in Figure 2, the gEUDs yielding 85% TCP (minimum) and the gEUDs yielding 99.99% TCP (maximum) were set accordingly as the lower and upper bounds for the sampling ranges for the three TVs in PSO. These gEUD values depend on the patient's individual TVs since the TCP model accounts for the structure's volume. Similarly, for OARs, the upper bound for the corresponding gEUD sampling range was defined by the gEUD yielding 10% NTCP, the maximum acceptable NTCP level in our investigation. These TCP and NTCP levels can be modified according to clinical requirements and can be set at different levels for different TVs or OARs if wished.

Treatment plans were calculated using Varian's Photon Optimizer (PO_15.6.04). Due to access restrictions in the Eclipse scripting application interface (ESAPI), only serial execution on CPUs for plan optimization was available. Dose volumes were computed using the Anisotropic Analytical Algorithm (AAA_15603), and the final DVH for the treatment plans was estimated using the DVH Estimation Algorithm (15.6.04).

The PSO parameterization included an inertia weight of 0.729, cognitive weight (c_1_) and social weight (c_2_) of 1.49445, an initial velocity attenuation of 0.1, and a particle reset probability of 0.001. The optimization was performed using 150 particles for 10 epochs, resulting in a total of 1500 iterations per patient case. A detailed description of the fine‐tuning is provided in the Supporting Information under the Fine‐Tuning PSO section. Source code of the PSO can be accessed from the GitHub repository [https://github.com/isachpaz/OptimizationSharp].

**FIGURE 3:**
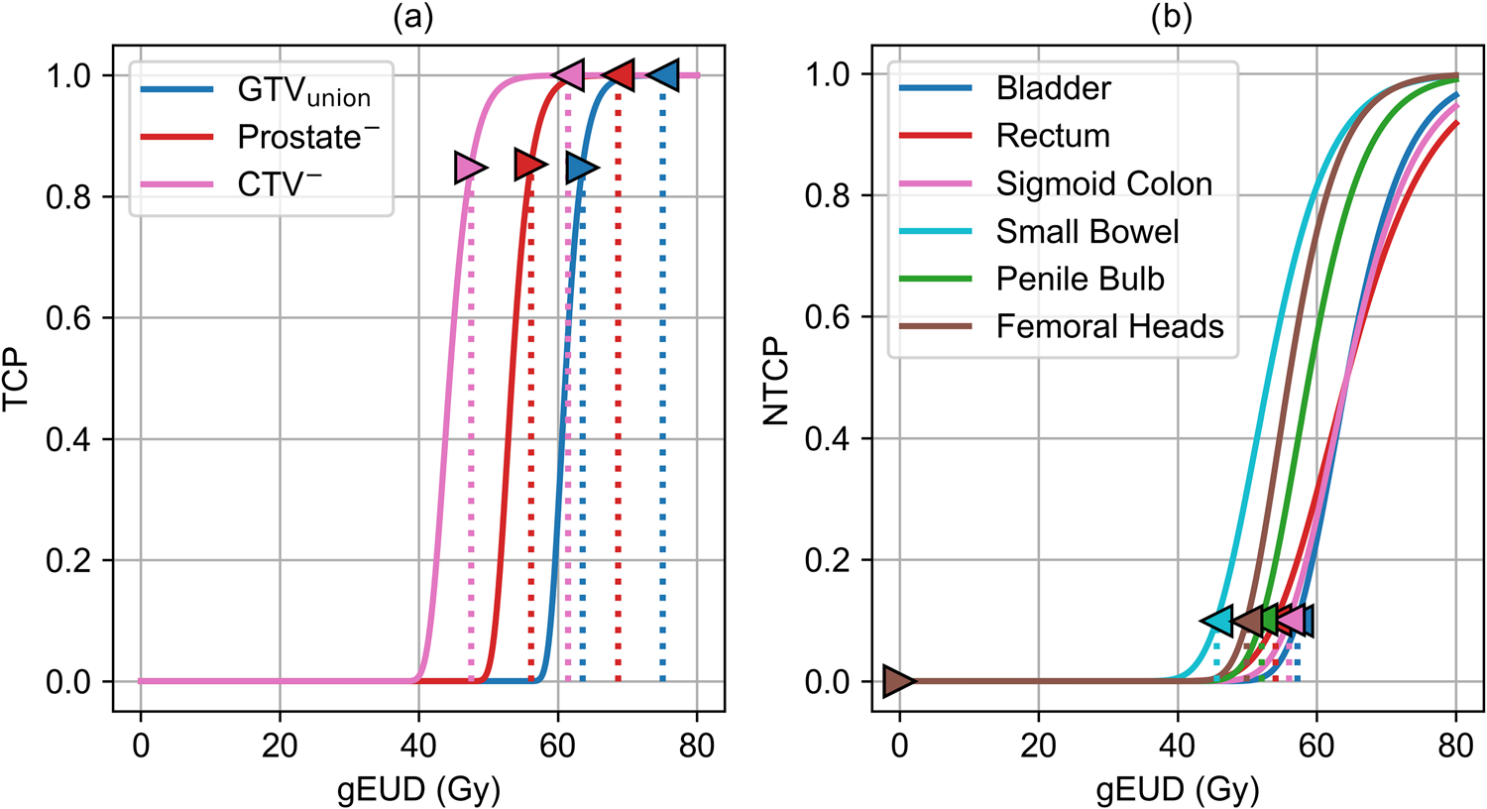
gEUD goal value sampling ranges for the different objective functions in PSO derived from the TCP, Equation (1), (a) and NTCP, Equation (4), (b) models for 20 treatment fractions. The dose response curves for GTV_union_, Prostate^−^ and CTV^−^have been calculated as an example using the volumes of patient case 1, of 2.76 cm^3^, 22.49 cm^3^ and 17.23 cm^3^, respectively (see Table 1). The resulting gEUD sampling ranges (triangles) for TCPs within 85% and 99.99% and NTCPs below 10%, acceptable TCP and NTCP levels considered in the study, are defined in the figure.

Considering the set x of VMAT optimizer variables, a solution $x^{*}\in X$ is then *Pareto non‐dominated* if

$$\begin{array}{cc} \forall x^{\prime }\in X, & x^{\prime }\neq x^{*}:P_{I}\left(x^{*}\right)\leq P_{I}\left(x^{\prime }\right)\wedge P_{B}\left(x^{*}\right)\geq P_{B}\left(x^{\prime }\right)\wedge \\ & \left[P_{I}\left(x^{*}\right)<P_{I}\left(x^{\prime }\right)\vee P_{B}\left(x^{*}\right)>P_{B}\left(x^{\prime }\right)\right] \end{array}$$

for the set of discovered solutions X. The set of all Pareto non‐dominated plans, that is, *Pareto optimal plans*, is the *Pareto optimal set*, which is defined in the parameter space. The remaining plans are called *Pareto dominated*. The image of the Pareto optimal set is called the *Pareto front*. The theoretical *Pareto global optimum* was determined by interpolating the Pareto front and defining the point with the highest *P_+_*. The Pareto global optimum is not an actually calculated Pareto optimal plan (*Pareto optimal solution*). If the solution space has been explored sufficiently, it is expected that the Pareto global optimum corresponds closely to the actually found solution with the highest *P_+_*, which was termed the *highest P_+_ plan* or *highest P_+_* solution. The *illusion point* (*utopia poin*t) is defined as $I=(\min\left(P_{I}\left(X\right)\right),\min\left(1-P_{B}\left(X\right)\right))$. If I lies far from the Pareto front, the Pareto front exhibits a strong trade‐off between *P_I_* and (1 − *P_B_*). Moving away from the highest *P_+_* solution on the Pareto front results in compromising either *P_I_* or *P_B_*.^40^ This concept is visualized in Figure 3.

### 2.5 Sensitivity analysis of prescription‐free treatment planning

To account for the variability of published *α/β* values and to evaluate thus the robustness of results, we followed the procedure described by Zamboglou et al.^37^ and Spohn et al.^22^ We defined two further parameter sets for the tumor *α/β* value of 1.2 and 2.7 Gy,^41^ and we generated prescription‐free treatment plans using those two additional parameter sets.

The remaining parameter values were estimated according to the following procedure: For each one of the *α/β* values, the parameter *α* of GTV_union_ was fitted, such that the resulting average TCP for the GTV_union_ using the fixed cell density value of 2.8·10^8^ cells/cm^3^ was the expected TCP of $\sqrt[{2}]{0.99}$ (see Supporting Information). The fitted *α* value was then used to estimate the ρ values for the remaining two TVs, Prostate^−^, and CTV^−^, as described previously in Section 2.3 (see also Supporting Information).

Four cases have been carefully selected (see Table 1) for the execution of the sensitivity analysis. To assess the similarity of the prescription‐free optimizations with the three TCP parameterizations the position and shape of the resulting Pareto fronts were visually inspected. The clinical dose constraints (Table 2) were evaluated for the Pareto optimal plans of each prescription‐free optimization. Additionally, a *γ*–analysis of the TVs’ dose distributions was performed using recommended criteria: a 3% dose difference, 2 mm distance‐to‐agreement, and a 10% dose threshold relative to the maximum dose. A *γ* passing rate of ≥ 95% was considered indicative of dosimetric equivalence, as suggested by Miften et al.^42^

## 3 RESULTS

### 3.1 TCP model parameter fitting

The final TCP model parameter values are listed in Table 4.

**TABLE 4:** Parameter values for the Poisson‐based TCP model (TCP–Parameter–Set 1).

Structure	*ρ*·10^5 ^(cells/cm^3^)	*α *(Gy^−1^)	*α/β *(Gy)
GTV_union_	2800	0.1205	1.6
Prostate^−^	5.4 [4.5, 6.9]	0.1205	1.6
CTV^−^	0.12 [0.10, 0.14]	0.1205	1.6

*Note*: Values in square brackets indicate 95% CI.

### 3.2 Prescription‐free radiobiology‐based radiation treatment planning

**FIGURE 4:**
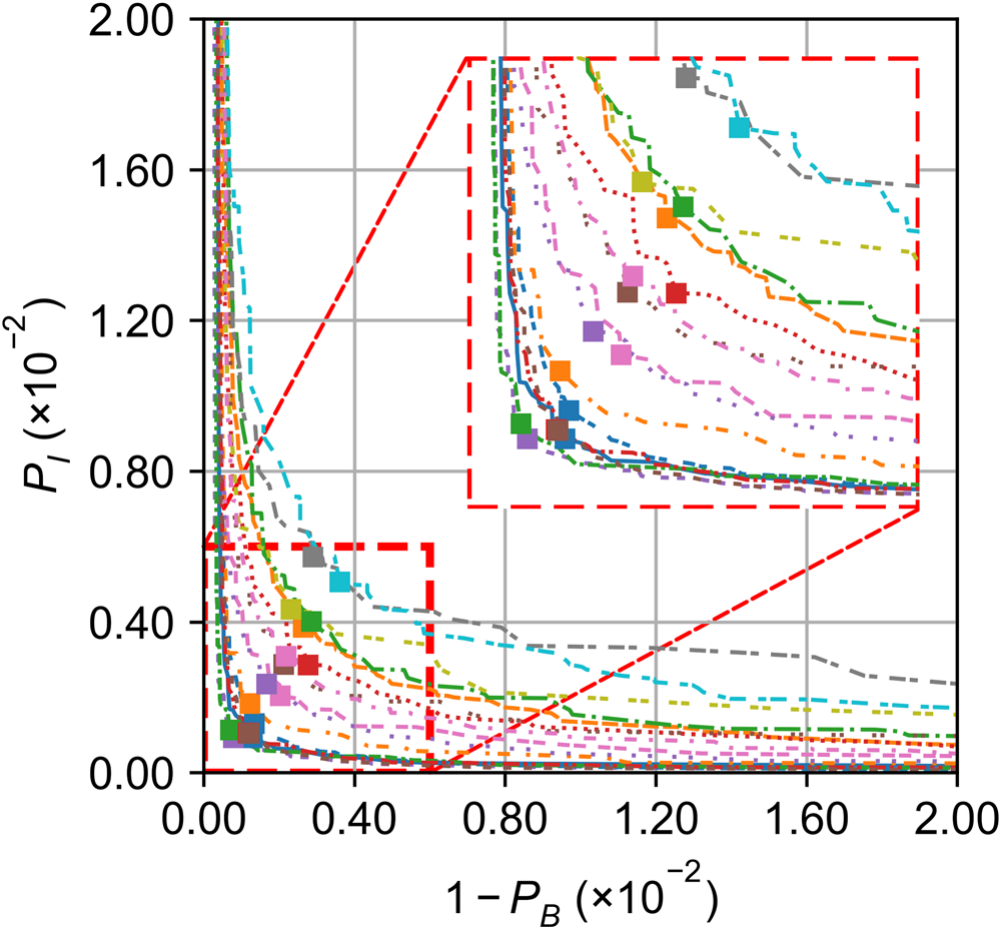
Pareto fronts of prescription‐free optimizations for all 17 patient cases and their highest *P_+_* plans marked as filled squares.

**FIGURE 5:**
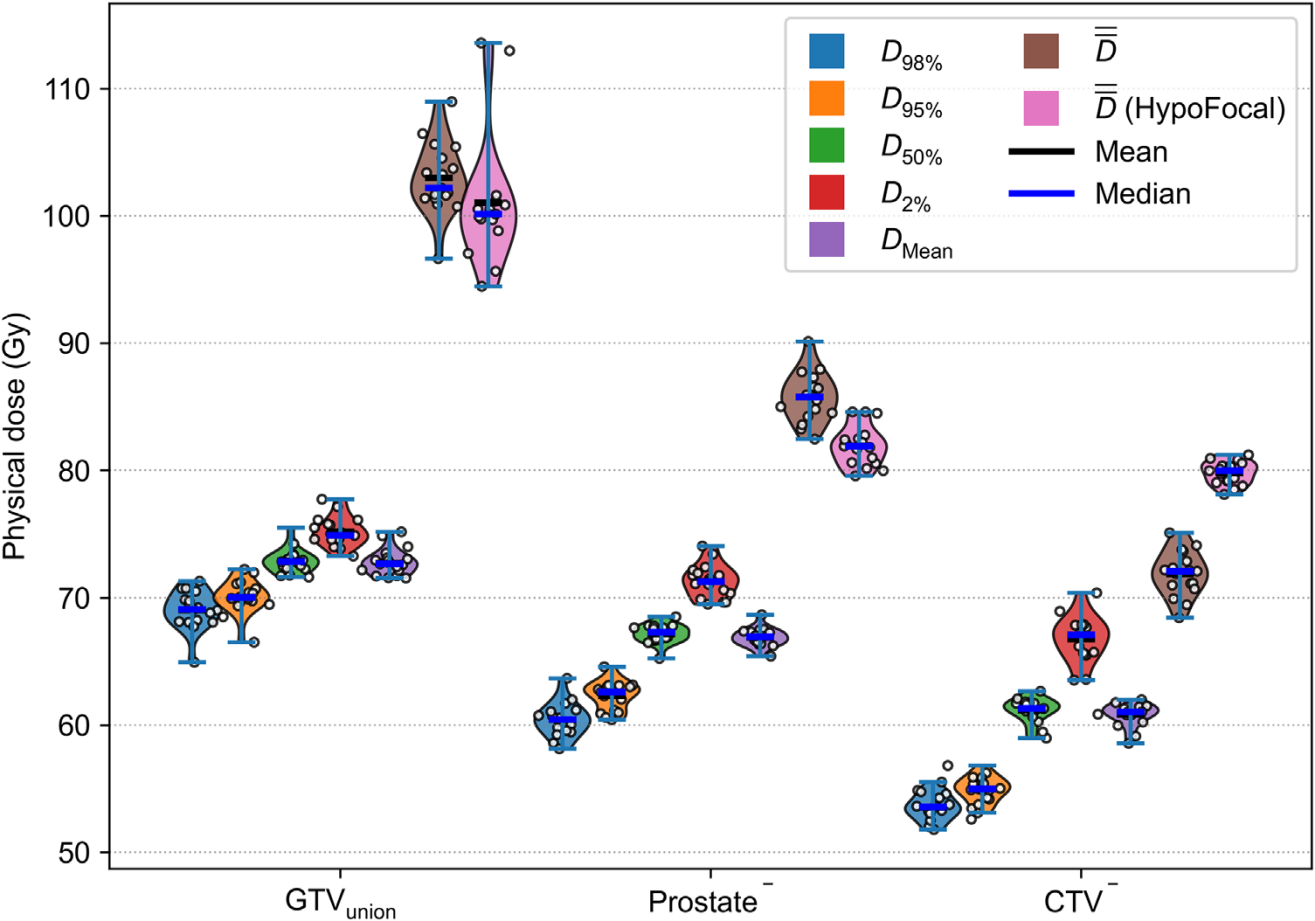
Dose–volume metrics of prescription‐free optimizations for all 17 patient cases. *D*_98%_, *D*_95%_, *D_50%_*, *D*_2%_, and *D*_Mean_ were calculated for the highest *P_+_* plans for 20 treatment fractions. $\bar{\bar{D}}$ values are given in EQD2 and were calculated using *α/β* = 1.6 Gy. One‐sided Wilcoxon signed‐rank tests were performed to compare $\bar{\bar{D}}$ between prescription‐free and HypoFocal plans. The analysis showed that $\bar{\bar{D}}$ was significantly greater for the prescription‐free than for HypoFocal plans in both GTV_union_ (*p* < 0.05) and Prostate^−^ (*p* < 0.01), and significantly lower in CTV^−^ (*p* < 0.01). Violin plots were generated using kernel density estimation with bandwidths selected by Scott's Rule.^43^

By applying the prescription‐free optimization method to all 17 patients, using the TCP model parameters listed in Table 4 and the NTCP model parameters in Table 3, we generated Pareto fronts in the bi–objective space of *P_I_* versus (1 − *P_B_*) (Figure 4). For each patient, the plan with the highest *P_+_* was selected as the optimal solution. The variability between the individual Pareto fronts is reflected in the variability of dose–volume metrics across patients, as illustrated in Figure 5. The generated Pareto fronts exhibit slight asymmetry. Due to this, the highest *P_+_* solutions do not align along a straight diagonal when plotted across patients, as it might be expected (see explanation of Figure 3). The TCP and NTCP values for those plans are listed in Table 5.

**TABLE 5:** Radiobiological metrics (TCP, *P_B_*, *P_I_*, and *P_+_*) for the highest *P_+_* plans obtained via prescription‐free optimization and for the corresponding HypoFocal plans across 17 patient cases.

	TCP of prescription‐free planning			TCP of HypoFocal trial			Prescription‐free planning			HypoFocal trial		
Case	$\mathrm{GT}V_{\mathrm{union}}$	$\mathrm{Prostat}e^{-}$	$\mathrm{CT}V^{-}$	$\mathrm{GT}V_{\mathrm{union}}$	$\mathrm{Prostat}e^{-}$	$\mathrm{CT}V^{-}$	$P_{B}$	$P_{I}$	$P_{+}$	$P_{B}$	$P_{I}$	$P_{+}$
1	99.90	99.88	99.93	99.90	99.77	99.99	99.71	0.04	99.67	99.66	2.10	97.57
2	99.94	99.71	99.96	99.76	99.60	99.99	99.61	0.18	99.43	99.35	4.39	94.99
3	99.94	99.75	99.96	99.92	98.69	99.98	99.65	0.24	99.41	98.59	2.13	96.49
4	99.82	99.80	99.92	99.77	99.25	99.99	99.55	0.13	99.42	99.01	2.44	96.60
5	99.95	99.91	99.96	99.83	99.74	100.00	99.82	0.04	99.79	99.56	3.20	96.37
6	99.86	99.82	99.95	99.80	99.36	99.99	99.63	0.14	99.50	99.15	3.20	95.97
7	99.92	99.83	99.94	99.06	99.83	99.99	99.69	0.09	99.60	98.89	2.64	96.28
8	99.63	99.81	99.93	99.79	99.41	99.99	99.38	0.35	99.03	99.20	3.61	95.62
9	99.93	99.76	99.83	99.28	99.71	99.99	99.53	0.27	99.26	98.99	1.07	97.93
10	99.80	99.48	99.95	98.57	99.45	99.99	99.23	0.29	98.94	98.01	3.31	94.77
11	99.94	99.84	99.92	99.91	99.29	99.99	99.71	0.06	99.65	99.19	2.48	96.73
12	99.87	99.80	99.95	99.66	99.63	99.99	99.62	0.08	99.54	99.28	3.26	96.04
13	99.93	99.95	99.94	100.00	99.78	99.99	99.82	0.06	99.77	99.77	3.19	96.58
14	99.88	99.94	99.93	99.80	99.88	100.00	99.74	0.05	99.69	99.68	4.25	95.45
15	99.96	99.87	99.88	100.00	99.30	99.99	99.71	0.09	99.62	99.28	3.07	96.24
16	99.94	99.92	99.88	99.88	99.38	99.99	99.75	0.05	99.71	99.25	3.16	96.11
17	99.94	99.73	99.91	99.77	99.47	99.99	99.59	0.15	99.43	99.23	3.68	95.58
Mean	99.89	99.81	99.93	99.69	99.50	99.99	99.63	0.14	99.50	99.18	3.01	96.20
Std	0.08	0.11	0.03	0.37	0.29	0.00	0.15	0.09	0.23	0.41	0.79	0.78
Median	99.93	99.82	99.93	99.80	99.47	99.99	99.65	0.09	99.54	99.23	3.19	96.24
Min	99.63	99.48	99.83	98.57	98.69	99.98	99.23	0.04	98.94	98.01	1.07	94.77
Max	99.96	99.95	99.96	100.00	99.88	100.00	99.82	0.35	99.79	99.77	4.39	97.93

*Note*: Values are given in percent.

Relevant dosimetric parameters for TVs of the highest *P_+_* plans are presented in Figure 5. The dose distributions of these optimal solutions were converted into their biologically iso‐effective uniform dose $\bar{\bar{D}}$ in EQD2. On average, the resulting $\bar{\bar{D}}$ was 102.81 Gy (±2.53 Gy) for the GTV_union_, 85.57 Gy (±1.79 Gy) for the Prostate^−^, and 71.96 Gy (±1.72 Gy) for the CTV^−^, respectively. The values in parentheses represent the standard deviation across the patient cohort. These uniform doses follow the same escalation pattern as the HypoFocal trial prescriptions (highest boost on the primary tumor volume, intermediate coverage of the prostate gland, and lowest dose to the wider CTV) while exceeding the corresponding HypoFocal EQD2 prescription levels (99.17 Gy for PTV3, 76.7 Gy for PTV2, and 57.50 Gy for PTV1, see Table 2). When comparing $\bar{\bar{D}}$ between the prescription‐free plans and the corresponding clinical HypoFocal plans, significant differences were observed for all three TVs. The prescription‐free plans yielded slightly higher $\bar{\bar{D}}$ values in GTV_union_ and Prostate^−^ (on average by 2–3 Gy), but markedly lower values in CTV^−^ (by more than 8 Gy; see Figure 5).

A quantitative comparison between the radiobiological measures of the prescription‐free optimized plans and those created for the HypoFocal trial (Plan1 of Spohn et al.^22^) by utilizing prescriptions is shown in Table 5. A series of paired one‐sided Wilcoxon signed‐rank tests was conducted to evaluate differences in TCP, *P_B_*, *P_I_*, and *P_+_* between prescription‐free and HypoFocal treatment plans. The analyses revealed that the prescription‐free method resulted in significantly higher TCP for the GTV_union_ (*p* < 0.01) and Prostate^−^ (*p* < 0.01), as well as significantly lower TCP for the CTV^−^ (*p* < 0.01). Additionally, significantly higher *P_B_* and significantly lower *P_I_* were observed for the prescription‐free plans, which consequently resulted in significantly higher *P_+_* values compared with the clinical HypoFocal plans (all *p* < 0.01). Specifically, prescription‐free optimization led to increased expected tumor control probability and reduced expected risk of normal tissue complications. Importantly, the observed increase in *P_+_*, representing the net therapeutic gain, is largely attributable to a substantial reduction in *P_I_*, which decreased from 3% to 0.14% across the cohort.

The prescription‐freely‐generated highest *P_+_* plans for all 17 cases successfully met all the dose–volume constraints defined in the HypoFocal trial for the 7 OARs (see Table 2). A summary of the obtained DVHs is provided in the Supporting Information.

**FIGURE 6:**
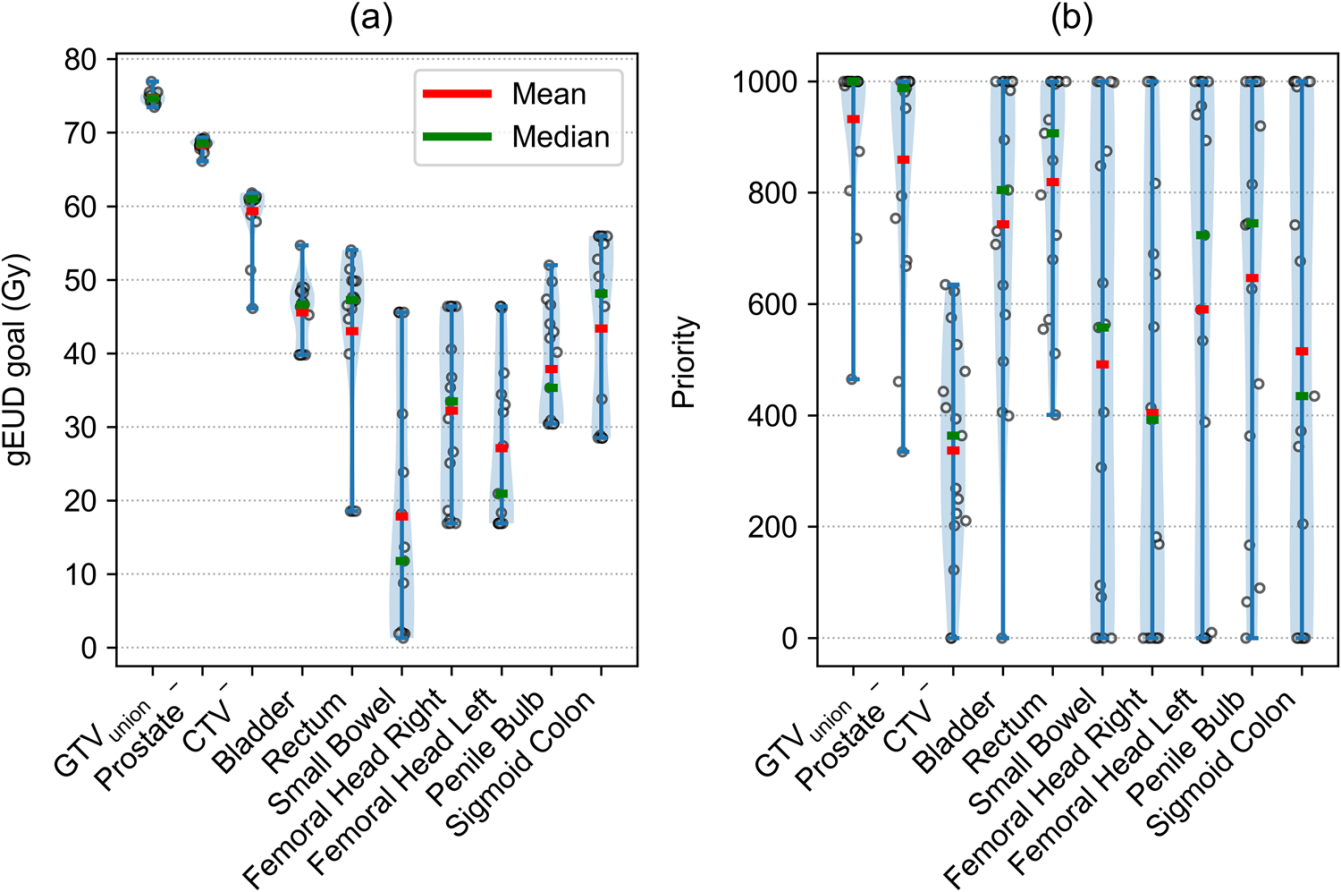
gEUD goal (a) and priority (b) values of the optimization objectives of the treatment plans with the highest *P_+_*. Violin plots were generated using kernel density estimation with bandwidths selected by Scott's Rule.^43^

The VMAT objectives, including gEUD goal values and priority values, for each highest *P_+_* plan are shown in Figure 6. As visible in this figure, the PSO optimizer genuinely explores the search space without being constrained by arbitrary limits.

The Homogeneity Index (*HI*), defined as (*D_2%_* − *D_98%_*)/*D_50%_*, and as recommended by ICRU Report 83^44^ has also been considered for comparing the dose distributions in the three TVs of the two plan categories. The mean (±standard deviation) *HI* for the GTV_union_, Prostate^−^, and CTV^−^ were 0.09 (±0.03), 0.29 (±0.05), and 0.27 (±0.09), respectively, for the HypoFocal plans, and 0.18 (±0(p.03), 0.32 (±0.07), and 0.49 (±0.10), respectively, for the prescription‐free plans. One‐sided paired Wilcoxon signed‐rank tests revealed that the prescription‐free approach resulted in significantly higher *HI* compared to HypoFocal plans for GTV_union_ (*p* < 0.01), Prostate^−^ (*p* < 0.05), and also for CTV^−^ (*p* < 0.01).

### 3.3 Sensitivity analysis

The fitted TCP model parameter values for the sensitivity analysis are listed in Table 6. For each of the four patients included in the sensitivity analysis, a prescription‐free optimization was performed using the two additional parameter value sets for the TCP model listed in Table 6. The NTCP parameter values remained unchanged and are listed in Table 3.

**TABLE 6:** Parameter value sets for the TCP model for the sensitivity analysis.

	TCP–parameter–set 2			TCP–parameter–set 3		
Structure	*ρ*·10^5^ (cells/cm^3^)	*α* (Gy^−1^)	*α/β* (Gy)	*ρ*·10^5^ (cells/cm^3^)	*α* (Gy^−1^)	*α/β* (Gy)
GTV_union_	2800	0.09631 [0.09390, 0.09758]	1.2	2800	0.16393 [0.16010, 0.16595]	2.7
Prostate^−^	3.11 [2.6, 4.0]			0.5 [4.2, 6.3]		
CTV^−^	0.07 [0.05, 0.08]			0.11 [0.1, 0.13]		

*Note*: Values in square brackets indicate 95% CI.

Visual inspection of the resulting Pareto fronts and optimization progress curves revealed no substantial deviations in shape or position across the sets (see Supporting Information). All clinical dose constraints for OARs (see Table 2) were satisfied by all the highest *P_+_* plans. These findings were supported by *γ*–analysis results, all meeting the recommended passing criteria.

## 4 DISCUSSION

The aim of this work was to adapt and extend the concept of prescription‐free, radiobiological treatment planning, first proposed by Källman et al.^10^ and later applied to prostate IMRT by Kim and Tomé,^11^ to the setting of modern VMAT for primary prostate cancer. To achieve this, we implemented a fully automated optimization framework within a commercial TPS (Eclipse) that replaced fixed physical dose prescriptions with prescription‐free planning in biological objective space.

In our framework, radiobiological response models were incorporated directly into VMAT optimization by computing TCP for three TVs and NTCP for seven OARs. For each VOI, clinically motivated TCP or NTCP levels were specified beforehand to restrict the optimization to clinically acceptable regions of the solution space. Because TCP, NTCP and the derived *P_+_* metric were calculated from the full DVH, they provided a consistent and biologically interpretable basis for evaluating tumor control and normal‐tissue effects across different treatment plans.

Using PSO with *P_+_* as the single objective, the framework automatically generated patient‐specific sets of Pareto‐optimal, clinically deliverable VMAT plans in the bi–objective space defined by *P_I_* and (1 − *P_B_*). These biological Pareto fronts allowed either selection of the plan with the highest *P_+_* or explicit exploration of trade‐offs between tumor control and OAR risk, rather than producing a single optimized solution. To our knowledge, this is the first implementation of a fully automated, prescription‐free VMAT optimization approach within a commercial TPS that yields Pareto fronts based solely on radiobiological objectives.

Zhao et al.^9^ and their earlier work,^8^, ^45^ utilize a dose‐painting‐by‐voxels strategy in which population‐based histology distributions are incorporated directly into the optimization. In contrast, our framework employed a dose‐painting‐by‐contours approach, as several studies have reported improved robustness of contour‐based strategies against spatial and temporal uncertainties in intraprostatic tumor biology.^46^ We defined three TVs (GTV_union_, Prostate^−^, and CTV^−^) and assumed homogeneous radiobiological parameters within each volume; GTV_union_ was selected as the union of PSMA–PET‐ and mpMRI‐derived GTVs, which has shown the highest overlap and sensitivity with histopathology.^47^ Furthermore, contour‐based dose painting avoids the sensitivity to atlas downscaling and voxel‐level noise reported for voxel‐based approaches such as those of Zhao et al.^45^

TCP parameters were fitted, including clonogenic cell densities of 2.8·10^8^, 5.4·10^5^, and 1.2·10^4^ cells/cm^3^ for GTV_union_, Prostate^−^, and CTV^−^_,_ respectively. These fitted clonogenic cell density values were also in line with previous reports. Van Lin et al.^48^ reported cell density values of 10^7^ cells/cm^3^ for the GTV and 10^5^ cells/cm^3^ for the rest of the prostate (Prostate^−^). Similar values were reported by Casares‐Magaz et al.^49^ based on ADC maps. In addition, our calculated cell density values for Prostate^−^, derived from histopathology data on intraprostatic satellite lesions, were in very good agreement with the fitted values presented in Table 4. Taken together, this concordance with both literature‐based and histopathology‐based estimates further supported the plausibility of the radiobiological parameters used in our study.

We applied the framework to 17 patients with unfavorable intermediate‐risk PCa. For each case, a patient‐specific biological Pareto front was generated, and the plan with the highest *P_+_* was selected for comparison with the corresponding HypoFocal plan. As shown in Table 5, the prescription‐free plans consistently achieved higher predicted tumor control and lower predicted normal‐tissue complication risks than the clinically generated HypoFocal plans. The dose distributions of the highest *P_+_* plans followed the same escalation pattern as the HypoFocal prescriptions (higher, intermediate and lower dose to GTV_union_, Prostate^−^ and CTV^−^, respectively) but exceeded the corresponding biologically iso‐effective uniform dose, $\bar{\bar{D}}$, levels for GTV_union_ and Prostate^−^, while remaining lower for CTV^−^, when compared to the clinical HypoFocal plans. The observed significantly higher *P_B_* and *P_+_* values and lower *P_I_* values for the prescription‐free method resulted from a different dose prescription pattern among the three TVs when compared with the clinical HypoFocal plans (see Table 5 and Figure 5). These results suggest that a slight to moderate dose escalation in GTV_union_ and Prostate^−^, in combination with a marked de‐escalation in CTV^−^ may lead to individualized treatment plans that are advantageous in terms of *P*_+_ compared to a standardized clinical protocol. Although statistical significance does not necessarily imply clinical meaningfulness, our results provide the indication that such individualized redistribution of dose delivery within the three TVs could be of clinical benefit.

As stated previously, we assume that, with the proposed framework, the solution space is sufficiently explored; therefore, the solution with the highest *P*_+_ is expected to approximate the Pareto global optimum. This assumption holds as long as all generated Pareto non‐dominated plans are not affected by local optima. The proposed framework is based on a heuristic approach involving the photon optimization algorithm (PO)^50^ and a stochastic PSO optimizer. Consequently, limitations related to local optima in PO may propagate into the explored Pareto front. Although PO is not a fully deterministic solver, this represents an intrinsic limitation of the proposed framework.

The prescription‐free plans exhibited reduced dose homogeneity across the three TVs compared with the HypoFocal plans, reflected by systematically higher *HI* values, particularly in CTV^−^. This behavior is consistent with radiobiological optimization, which prioritizes tumor control and normal‐tissue sparing over enforcing uniform dose distribution.^51^ To mitigate excessive heterogeneity associated with the dose‐painting‐by‐contours approach, lower and upper gEUD objectives with high absolute *a*‐parameters (±40) were included for all TVs.

The robustness of the framework was further supported by the sensitivity analysis performed with two additional TCP parameter sets (Table 6). Across all examined cases, the resulting Pareto fronts showed no meaningful deviations in shape or position, and the highest *P_+_* plans consistently satisfied all clinical OAR dose constraints. These findings were reinforced by *γ*‐Analysis of the TV dose distributions, which demonstrated dosimetric agreement within recommended criteria. Taken together, this indicates that the prescription‐free optimization is stable with respect to plausible variations in TCP model parameters and does not exhibit susceptibility to parameter‐induced distortions in dose distribution or plan quality.

The proposed prescription‐free framework already incorporates several patient‐specific biological characteristics through multi‐modality biological image‐based definition of tumor volumes, target volume‐dependent clonogenic cell density distributions and TCP modelling. These elements account for inter‐patient variability in anatomy and tumor burden, which strongly influence the predicted biological response. Nevertheless, a remaining limitation is that intrinsic radiosensitivity and cell density parameters were assumed to be population‐based and homogeneous within tumor regions (TVs). Emerging approaches, such as the intrinsic radiosensitivity index, derive tumor radiosensitivity from gene expression profiles and integrate this information within LQ modelling to estimate tumor biological response.^52^, ^53^ Furthermore, clonogenic cell density distributions may be derived from ADC maps, as reported by Casares‐Magaz et al.^49^ Incorporation of such patient‐specific radiosensitivity and cell density surrogates could further individualize TCP and NTCP estimation within the prescription‐free optimization framework as these models mature and become clinically validated.

A direct numerical comparison of OAR doses between the prescription‐free and HypoFocal plans should be interpreted with caution because the two planning strategies differ fundamentally in how target volumes were defined. The HypoFocal plans were optimized on PTVs that incorporated 2–6 mm setup margins, whereas our prescription‐free optimization was applied directly to CTVs and GTV_union_ without additional margins. As a result, the geometric proximity between targets and adjacent OARs was inherently greater in the HypoFocal plans, leading to systematically higher OAR doses and correspondingly elevated predicted NTCP values. In our patient cohort, IGRT with daily CBCT‐to‐CT registration and six‐degree‐of‐freedom corrections rendered systematic setup errors negligible, consistent with beam–imager isocenter deviations ≤0.5 mm on the stereotactic treatment delivery system. Under these conditions, residual uncertainty is dominated by intrafractional prostate motion, which constitutes a random error. Jin et al.^54^ demonstrated that random errors translate the TCP curve toward higher doses without principally altering its shape when systematic errors are low (≤2.0 mm); therefore, the tumor‐volume–related dose levels inferred from our prescription‐free plans remain valid in this IGRT setting.

In the current implementation, VMAT optimizations for candidate plans are executed sequentially due to TPS constraints: the ESAPI interface does not support concurrent VMAT optimizer runs or GPU acceleration. Consequently, the average runtime is 72 ±20 s per plan, yielding a total of ∼30 h per patient for prescription‐free optimization (150 particles · 10 epochs). Prior work has shown large speedups with GPU utilization; for example, Spalding et al. (2020)^55^ reported an 84% reduction in computation time for prostate plans with a hybrid CPU/GPU algorithm. Applying this factor to our workload implies a per‐plan time of 72 s · 0.16 = 11.52 s. If the 150 particles were evaluated in parallel, the wall‐clock time per epoch would be ∼11.5 s, and the 10‐epoch total would be ∼115 s (∼1.9 min) per patient in the ideal case. Thus, while the present implementation is computationally demanding, this is a limitation of the current TPS infrastructure, not of the framework itself; with GPU‐enabled optimization support, near‐real‐time clinical deployment would be achievable.

Future work will focus on two major directions. First, we aim to incorporate intrafractional motion and setup uncertainties directly into the prescription‐free optimization, building on our previously published modelling studies.^21^, ^22^ This will require probabilistic dose accumulation and scenario‐based evaluation to account for random error and its impact on TCP and NTCP. Second, we plan to extend and validate the framework across additional tumor sites to assess its generalizability and clinical robustness beyond prostate cancer. These efforts will help determine the broader applicability of biologically driven, prescription‐free VMAT planning in routine radiotherapy workflows.

## 5 CONCLUSION

The prescription‐free, radiobiology‐guided optimization framework presented in this study provides a promising alternative to conventional, prescription‐dependent radiotherapy planning methods. By directly considering a radiobiological objective function (*P_+_*) using PSO within predefined, patient‐specific and clinically acceptable TCP and NTCP levels, the framework generated highly individualized and clinically deliverable VMAT plans. A key innovation lies in the automatic generation of Pareto‐optimal, non‐dominated treatment plans in the bi–objective radiobiological space *P_I_* versus (1 − *P_B_*), enabling explicit visualization and quantitative assessment of trade‐offs between tumor control and normal tissue complication risk.

Compared to established clinical planning protocols, the generated plans achieved higher predicted tumor control, particularly for target volumes of high biological relevance and reduced normal tissue complication probabilities. Sensitivity analysis demonstrated robustness of the framework with respect to uncertainties in TCP model parameters.

Although geometric uncertainties were not yet incorporated, and the implementation remains computationally intensive due to sequential optimization, these limitations are not inherent and can be addressed in future developments. Planned extensions include integration of motion modelling, computational acceleration through parallelization and GPU support, and validation in additional tumor entities.

## CONFLICT OF INTEREST STATEMENT

The authors have no conflicts to disclose.

## CONSENT STATEMENT

Written informed consent was obtained from all participants prior to their inclusion in the study. All authors reviewed the final version of the manuscript and consent to its publication.

## DECLARATION OF GENERATIVE AI AND AI‐ASSISTED TECHNOLOGIES IN THE WRITING PROCESS

During the preparation of this work the authors used OpenAI's ChatGPT in order to improve language and readability. After using this tool/service, the authors reviewed and edited the content as needed and take full responsibility for the content of the publication.

## Supporting information




**Supporting File**: mp70347‐sup‐0001‐SuppMat.docx

## Data Availability

The datasets generated and/or analyzed during the current study are not publicly available due to ethical and privacy restrictions but are available from the corresponding author upon reasonable request.
