# Supplementary material for: A prescription‐free, radiobiology‐based framework for automated VMAT planning: A feasibility study in primary prostate cancer radiotherapy
Source: Med Phys. 2026 Feb 26;53(3):e70347. doi: 10.1002/mp.70347 (PMC12940459; doi:10.1002/mp.70347)
Supplement: Supplementary file 1 — Supporting File: mp70347‐sup‐0001‐SuppMat.docx [file MP-53-0-s001.docx]

Supplementary Material

# Radiobiological Modelling

The biologically iso-effective uniform dose $D$ is defined as the uniform dose that would produce the same biological effect as a non-uniform one. $D$is commonly defined as EQD2.

For target volumes $D$ was calculated for the Poisson TCP model (see Eq. 1 of the Manuscript) based on the assumption $TCP\left( D \right)=TCP\left( \vec{D} \right)$ for the given non-uniform dose distribution $\vec{D}$ as follows:

$$TCP\left( D \right)= e^{-\rho\cdot V\cdot e^{-\alpha\cdot D\cdot\left( 1+\frac{2}{\alpha/\beta} \right)}}= TCP\left( \vec{D} \right)$$

$$\Longleftrightarrow\ln\left( TCP\left( \vec{D} \right) \right)= -\rho\cdot V\cdot e^{-\alpha\cdot D\cdot\left( 1+\frac{2}{\alpha/\beta} \right)}$$

$$\Longleftrightarrow\ln\left( -\ln\left( TCP\left( \vec{D} \right) \right) \right)=\ln\left( \rho\cdot V \right)-\alpha\cdot D\cdot\left( 1+\frac{2}{\alpha/\beta} \right)$$

thus:

$$D=\frac{\ln\left( \rho\cdot V \right)-\ln\left( -ln(TCP\left( \vec{D} \right) \right)}{\alpha\cdot\left( 1+\left( \frac{2}{\alpha/\beta} \right) \right)}$$

For OARs and based on the Relative Seriality (RS) NTCP model (see Eq. 3 of the Manuscript) by assuming$NTCP\left( \vec{D} \right)=NTCP\left( D \right)$, $D$ is calculated as follows:

$$NTCP\left( D \right)= \left[ 1-\left( 1-P\left( D \right)^{s} \right) \right]^{1/s}=P\left( D \right) = NTCP\left( \vec{D} \right)$$

Using Eq. 4 of the Manuscript for $P\left( D \right)$ we have

$$NTCP\left( \vec{D} \right)=\exp\left( {-e}^{e\gamma-\left( \frac{D}{D_{50}} \right)\cdot\left( e\gamma-\ln\ln2 \right)} \right)$$

$$\Longleftrightarrow-ln \left( NTCP\left( \vec{D} \right) \right)=e^{e\gamma-\left( \frac{D}{D_{50}} \right)\cdot\left( e\gamma-\ln\ln2 \right)}$$

$$\Longleftrightarrow\ln\left( -\ln\left( NTCP\left( \vec{D} \right) \right) \right)=e\gamma-\left( \frac{D}{D_{50}} \right)\cdot\left( e\gamma-\ln\ln2 \right)$$

And finally

$$D=\frac{e\gamma-ln \left( -\ln\left( NTCP\left( \vec{D} \right) \right) \right)}{e\gamma-\ln\ln2}\cdot D_{50}$$

Where *D_50_* is also defined as EQD2.

# TCP model parameter fitting

Groen et al (2022)^1^ included 264 patients in a publication from the FLAME study, who were irradiated in 35 fractions with a simultaneous integrated boost (SIB) on the gross tumour volume (GTV). In Figure 2 of their article, they reported the predicted probability of local failure in dependence of near minimum dose (*D_98%_*) to the GTV for a median follow up time of 72 months. We converted the doses to EQD2 for 35 fractions using an *α/β* ratio of 1.6 Gy, and we fitted an exponential function to these data to derive a numerical model to predict probability of local failure *f*:

$$f\left( x \right)=a\cdot\exp\left( b\cdot x \right)$$

with *a* = 3.11 · 10^6^ with a 95% confidence interval of [2.402 · 10^6^, 3.818 · 10^6^] and b = -0.1705 Gy^-1^ with a 95% confidence interval of [-0.1735, -0.1675] and *x* the dose in EQD2.

Using the HypoFocal study prescription of 70 Gy in 20 fractions to GTV, we estimated an expected *P_B_* of 0.99 based on the above equation. Assuming an equal TCP for GTV and CTV and setting the expected local control probability in CTV⁻ to 1.0, $TCP_{CTV^{-}}$ = 1.0, we have:

$$P_{B}=0.99=TCP_{\mathrm{GT}V_{\mathrm{union}}}\cdot TCP_{\mathrm{Prostat}e^{-}}\cdot TCP_{\mathrm{CT}V^{-}}$$

with

$$TCP_{\mathrm{GT}V_{\mathrm{union}}}=TCP_{\mathrm{Prostat}e^{-}}= \sqrt{0.99}=0.995$$

For the subsequent steps, we considered the HypoFocal clinical treatment plans of the 17 cases included in our study (see Table 1 of the Manuscript).

We assumed the same radiosensitivity for all three target volumes and thus the same LQ-model parameters; *a* = 0.1205 Gy^-1^, *α/β* = 1.6 Gy and the cell density for GTV_union_ was fixed to 2.8 · 10^8^ cells/cm³ as in Spohn et al. (2020)^2^. Then we fitted the cell densities for the remaining TVs Prostate⁻ and CTV⁻ based on the TCP values calculated from the corresponding differential dose–volume histograms (DVH) by minimizing the following cost-functions:

$$\min_{\rho_{Prostate^{-}}} \left( \sqrt[2]{0.99}-\frac{1}{17}\sum_{i=1}^{17} TCP_{Prostate^{-}, i} \right)^{2}$$

$$\min_{\rho_{CTV^{-}}} \left( 1.0-\frac{1}{17}\sum_{i=1}^{17} TCP_{CTV^{-}, i} \right)^{2}$$

For patient case *i* of a total of the 17 patients. The Nelder-Mead-Simplex optimizer from the MathNet.Numerics library version 5.0.0 was used for that purpose.

# Fine-Tuning PSO

Four PSO parameterizations were selected to evaluate their impact on prescription-free planning, summarized in Table S1. For each parameterization, the optimization progress is visualized in Figure S1. It was observable that all of them reached comparable highest *P_+_* solutions with PSO-Parameter-Set 1 reaching its maximum earliest in two out of four cases. The associated Pareto fronts displayed in Figure S2 confirmed these observations.

Table S1: PSO parameterizations for prescription-free planning. *PSO Parameters from Künzel et al. (2020)^3^

| PSO-Parameter-Set | $\boldsymbol{1}$ | 2 | 3 | 4* |
| --- | --- | --- | --- | --- |
| Number of particles | 150 | 200 | 250 | 200 |
| Inertia Weight | 0.729 | 0.729 | 0.729 | 1.4 |
| C1 Cognitive Weight | 1.49445 | 1.49445 | 1.49445 | 2 |
| C2 Social Weight | 1.49445 | 1.49445 | 1.49445 | 2 |
| Velocity Initial Attenuation | 0.1 | 0.1 | 0.1 | 0.1 |
| Particle Reset Probability | 0.001 | 0.001 | 0.001 | 0.001 |


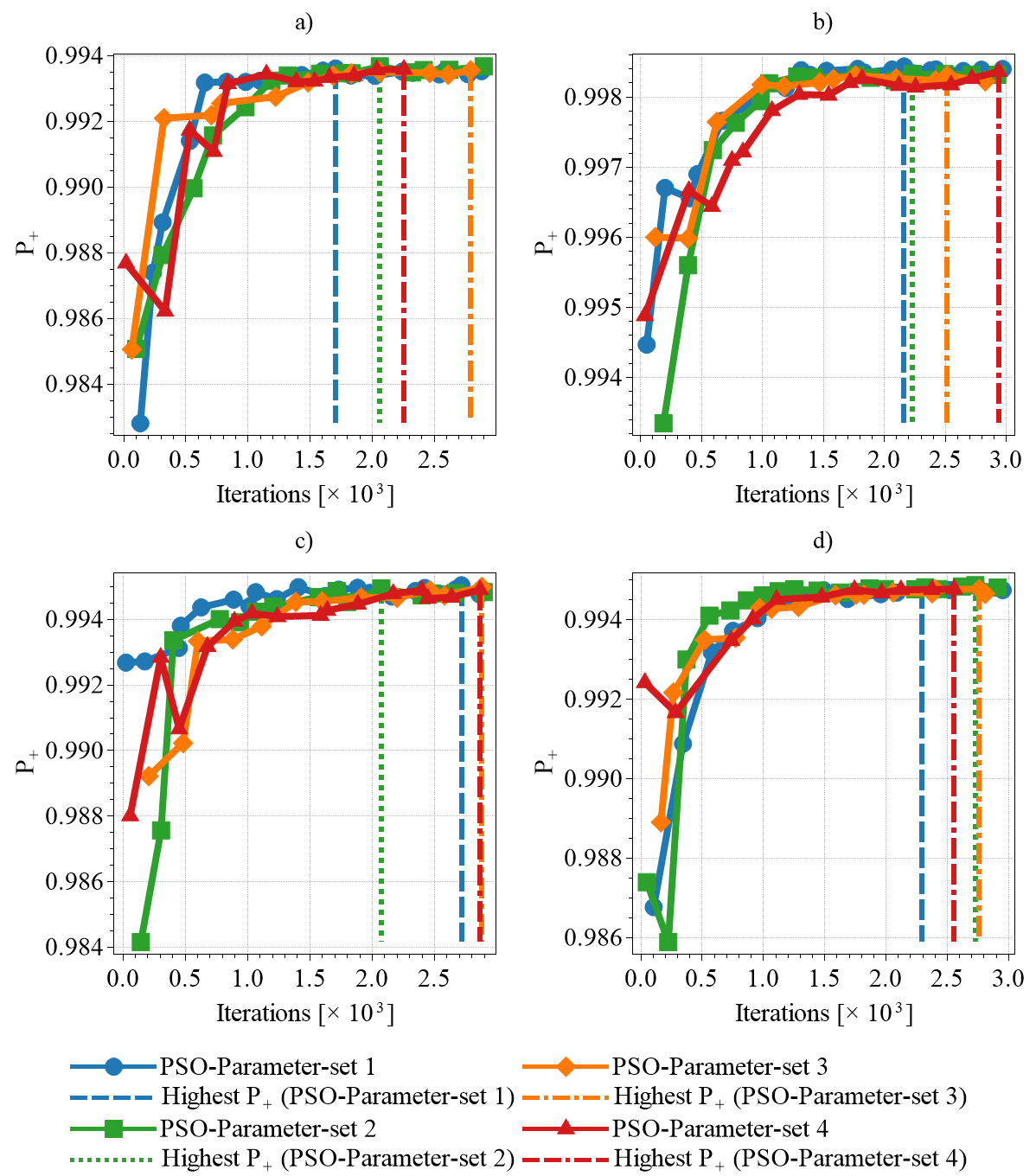


Figure S1: Progress of prescription-free optimizations for patient cases 2 (a), 5 (b), 6 (c) and 17 (d) and the four PSO parameterizations.


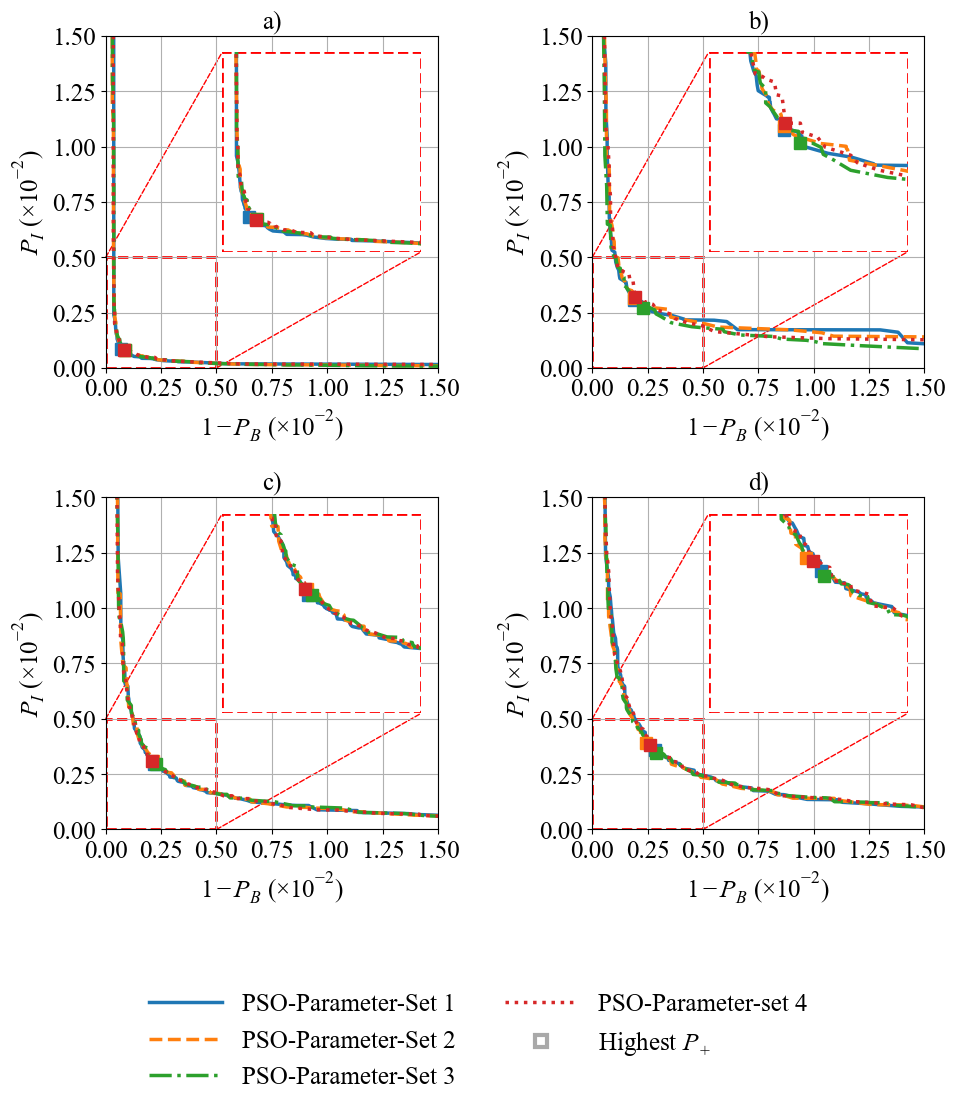


Figure S2: Pareto fronts of prescription-free optimizations for patient cases 2 (a), 5 (b), 6 (c) and 17 (d) and the four PSO parameterizations.

Secondly, we set the maximum number of iterations to 1500, re-executed the prescription-free planning with the four PSO parameterizations for the four patients and compared the resulting progress curves (Figure S3) and Pareto fronts (Figure S4) with the initial experiments, observing no significant difference.


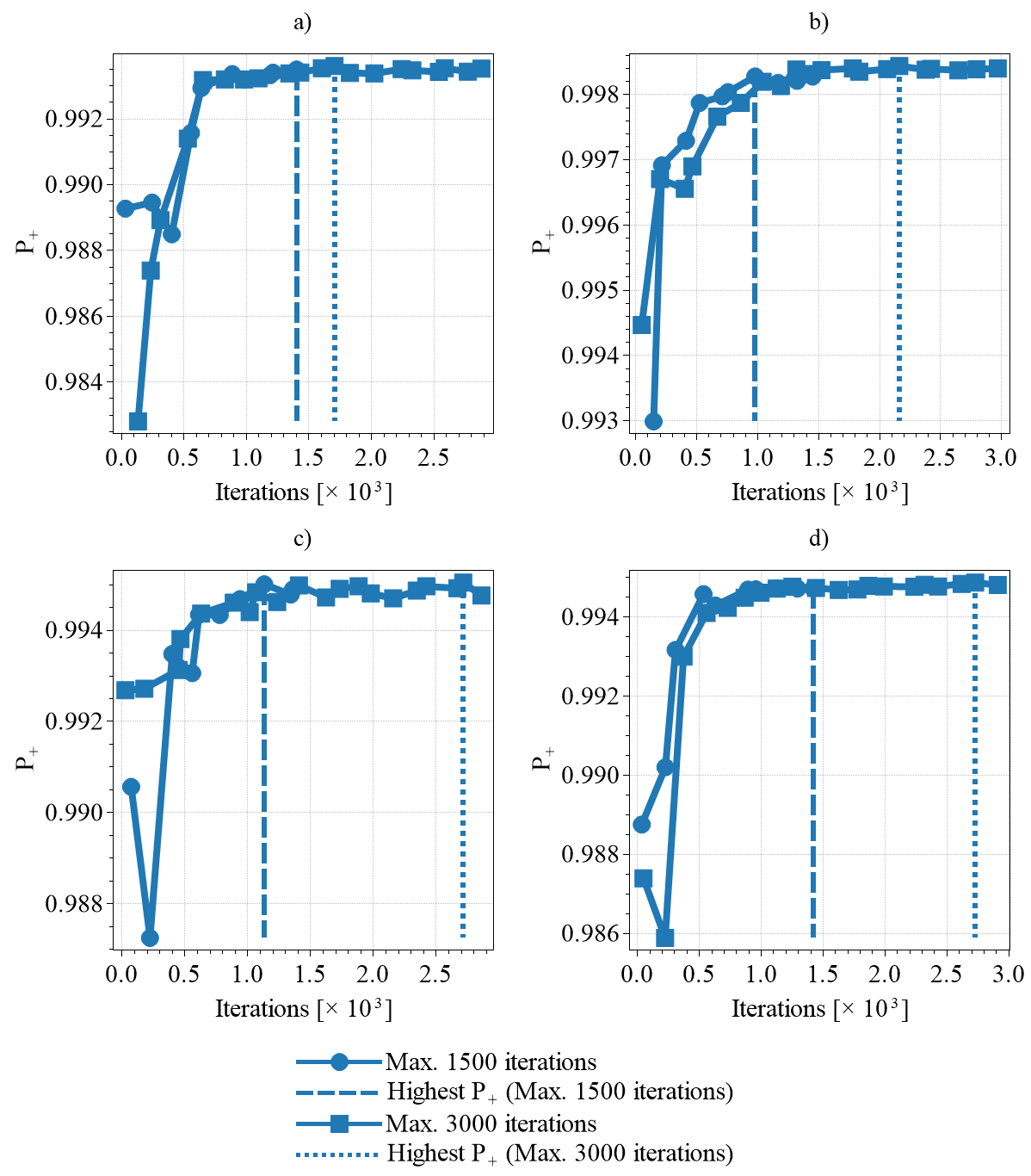


Figure S3: Progress of prescription-free optimizations for patient cases 2 (a), 5 (b), 6 (c) and 17 (d) with PSO-Parameter-Set 1 and maximum 3000 iterations versus maximum 1500 iterations per optimization.


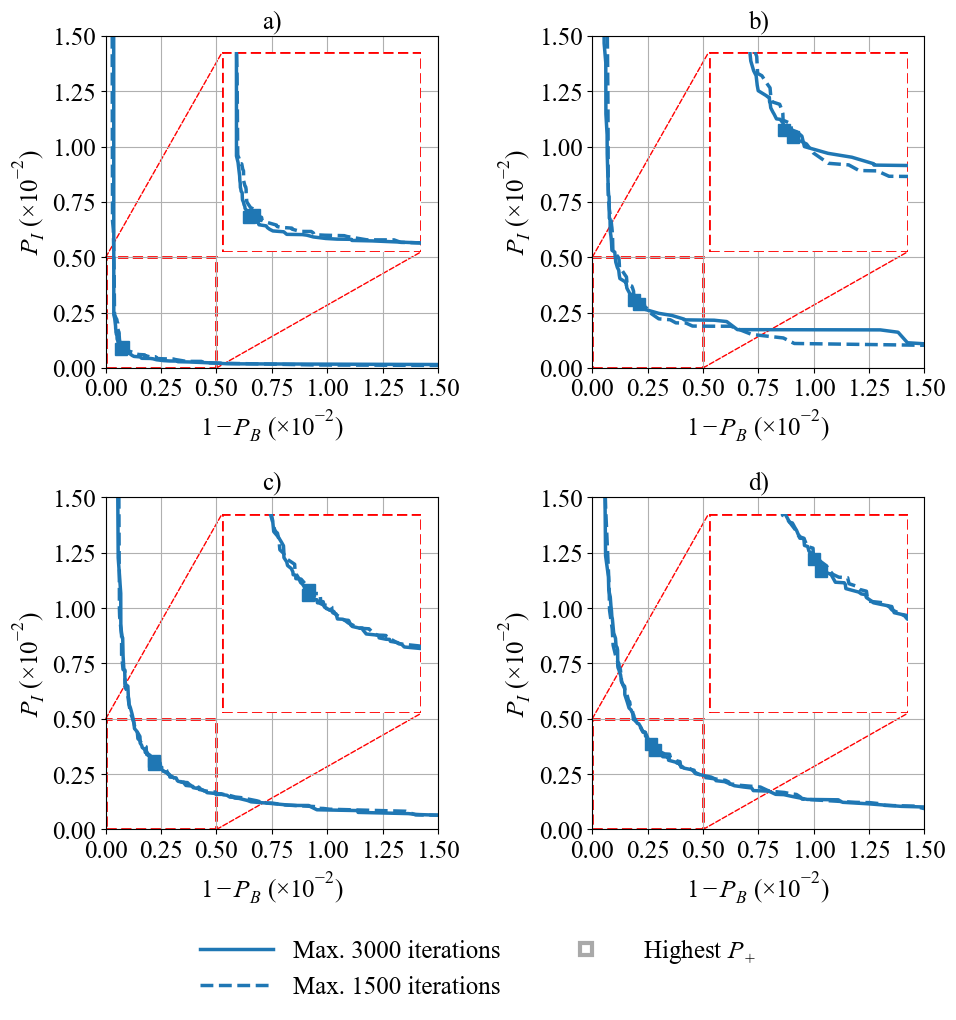


Figure S4: Pareto fronts of prescription-free optimizations for patient cases 2 (a), 5 (b), 6 (c) and 17 (d) with PSO-Parameter-Set 1 and maximum 3000 iterations versus maximum 1500 iterations per optimization.

In conclusion we selected the PSO-Parameter-Set 1 with a maximum of 1500 iterations to be the default configuration for prescription-free planning.

# Sensitivity Analysis of TCP model parameterizations

To evaluate the robustness of prescription-free planning with respect to variations in the underlying TCP model parameters, we conducted prescription-free optimizations for the four selected patients (Table 1 in the Manuscript) using the three TCP parameter sets defined in Tables 4 and 5 of the Manuscript. The resulting optimization progress curves and Pareto fronts (see Figure S5 and Figure S6) did not show substantial deviation in shape or position.

To further assess the similarity of the resulting dose distributions across the different TCP parameterizations, a *γ*-Analysis was performed. In accordance with the recommendations by Miften et al. (2018)^4^, a *γ* passing rate of ≥ 95% was considered acceptable, applying a dose difference criterion of 3%, a distance-to-agreement criterion of 2 mm, and a 10% dose threshold relative to the maximum dose within the three TVs. As shown in Table S2, all *γ* passing rates met or exceeded the 95% threshold for all patient cases and parameterizations.

All highest *P_+_* plans satisfied all clinical dose constraints for the OARs (see Table 2 of the Manuscript). In summary, the results demonstrated robustness of the prescription-free treatment planning method with respect to TCP model parameter uncertainty.


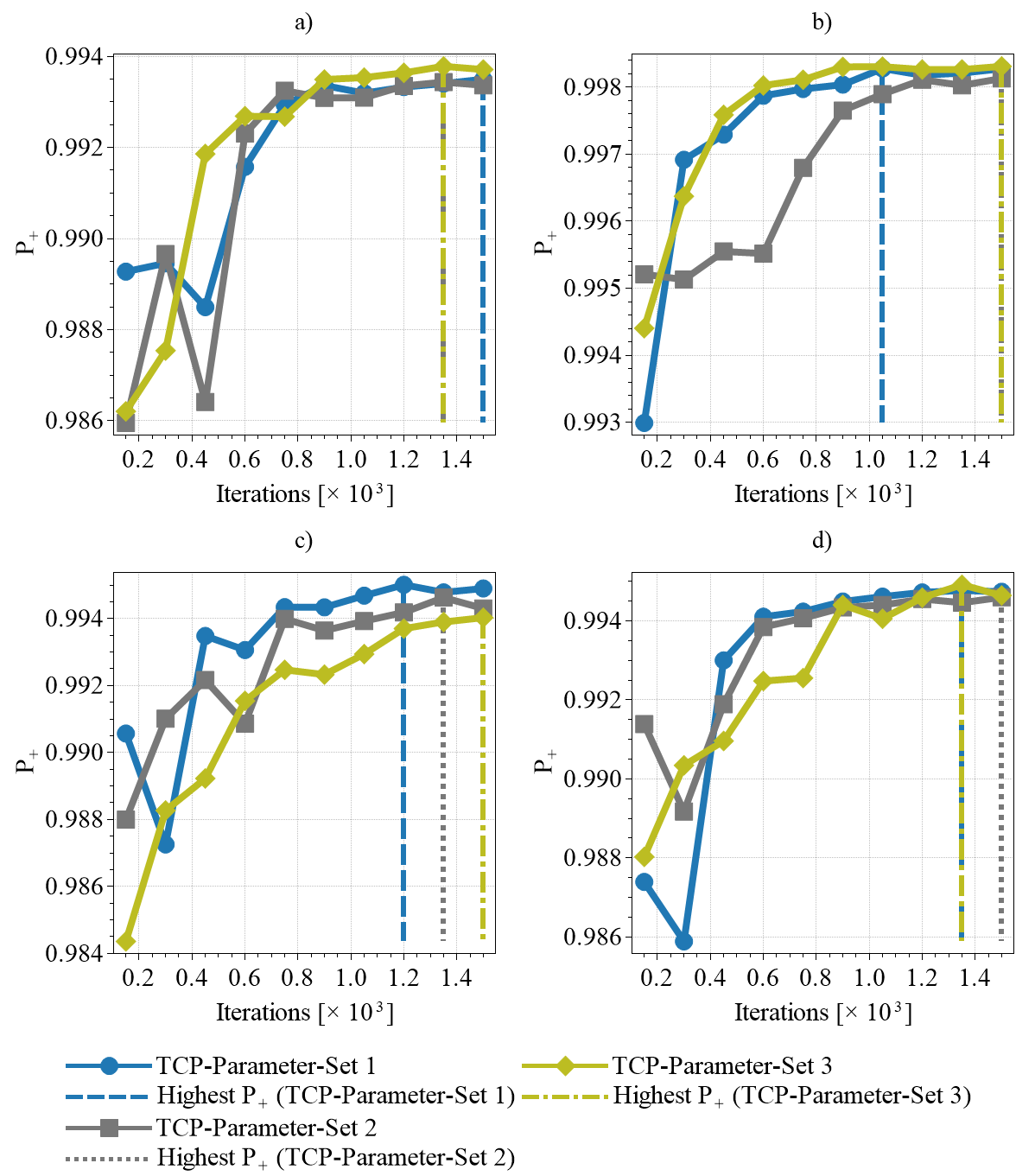


Figure S5: Progress of prescription-free optimizations for patient cases 2 (a), 5 (b), 6 (c) and 17 (d) with PSO-Parameter-Set 1 and three different TCP parameter sets for sensitivity analysis.


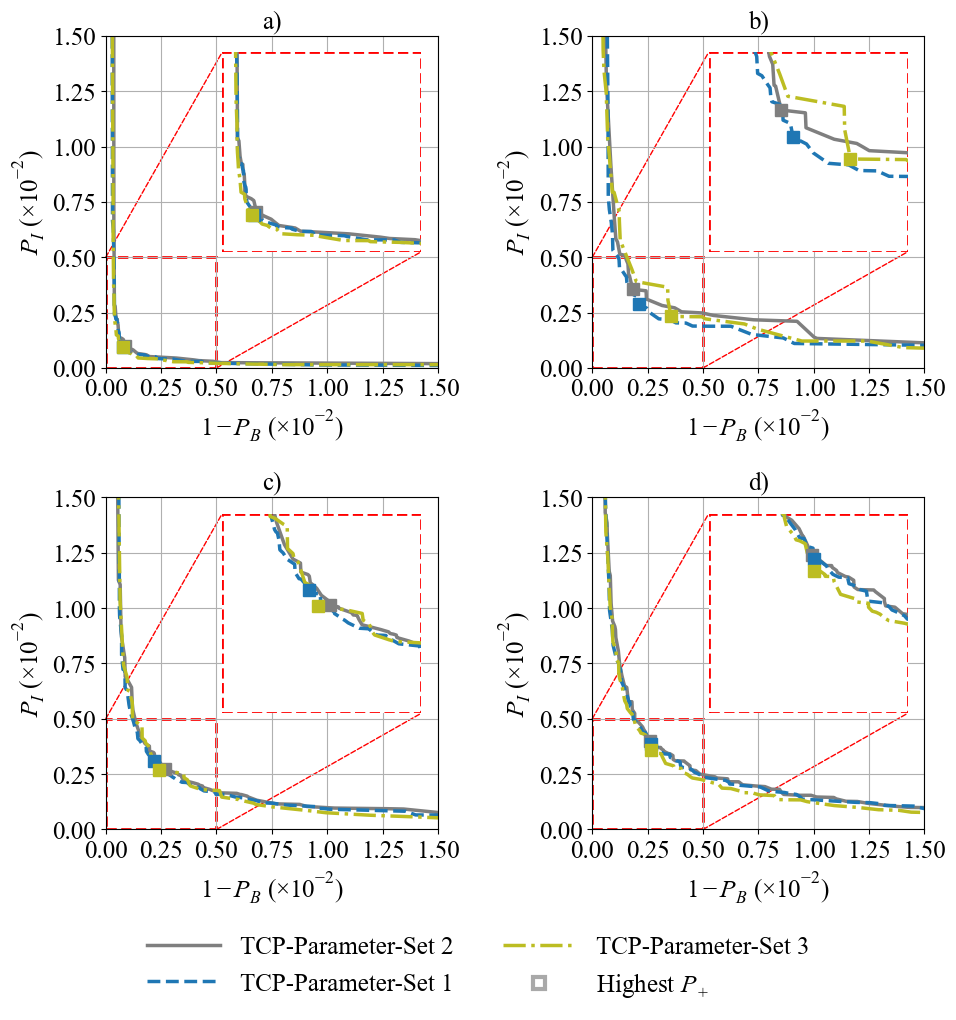


Figure S6: Pareto fronts of prescription-free optimizations for patient cases 2 (a), 5 (b), 6 (c) and 17 (d) with PSO-Parameter-Set 1 and three different TCP parameter sets for sensitivity analysis.

Table S2: *γ*-Analysis results of the TCP model sensitivity analysis.

|  | **Passing rate [%]** | | | **TCP-Parameter-Set** | |
| --- | --- | --- | --- | --- | --- |
| **Case** | **GTV_union_** | $\mathbf{Prostat}\mathbf{e}^{\mathbf{-}}$ | $\mathbf{CT}\mathbf{V}^{\mathbf{-}}$ | **Reference dose distribution** | **Evaluated dose distribution** |
| 2 | 100.00 | 99.95 | 99.92 | 1 | 2 |
|  | 99.71 | 99.74 | 99.84 | 1 | 3 |
|  | 100.00 | 99.95 | 99.76 | 2 | 3 |
| 5 | 100.00 | 99.94 | 95.25 | 1 | 2 |
|  | 100.00 | 99.44 | 100.00 | 1 | 3 |
|  | 100.00 | 99.61 | 97.44 | 2 | 3 |
| 6 | 100.00 | 99.93 | 99.93 | 1 | 2 |
|  | 98.83 | 95.26 | 95.41 | 1 | 3 |
|  | 100.00 | 95.93 | 95.83 | 2 | 3 |
| 17 | 100.00 | 99.71 | 98.78 | 1 | 2 |
|  | 99.33 | 98.79 | 99.39 | 1 | 3 |
|  | 100.00 | 99.91 | 99.94 | 2 | 3 |

# Results


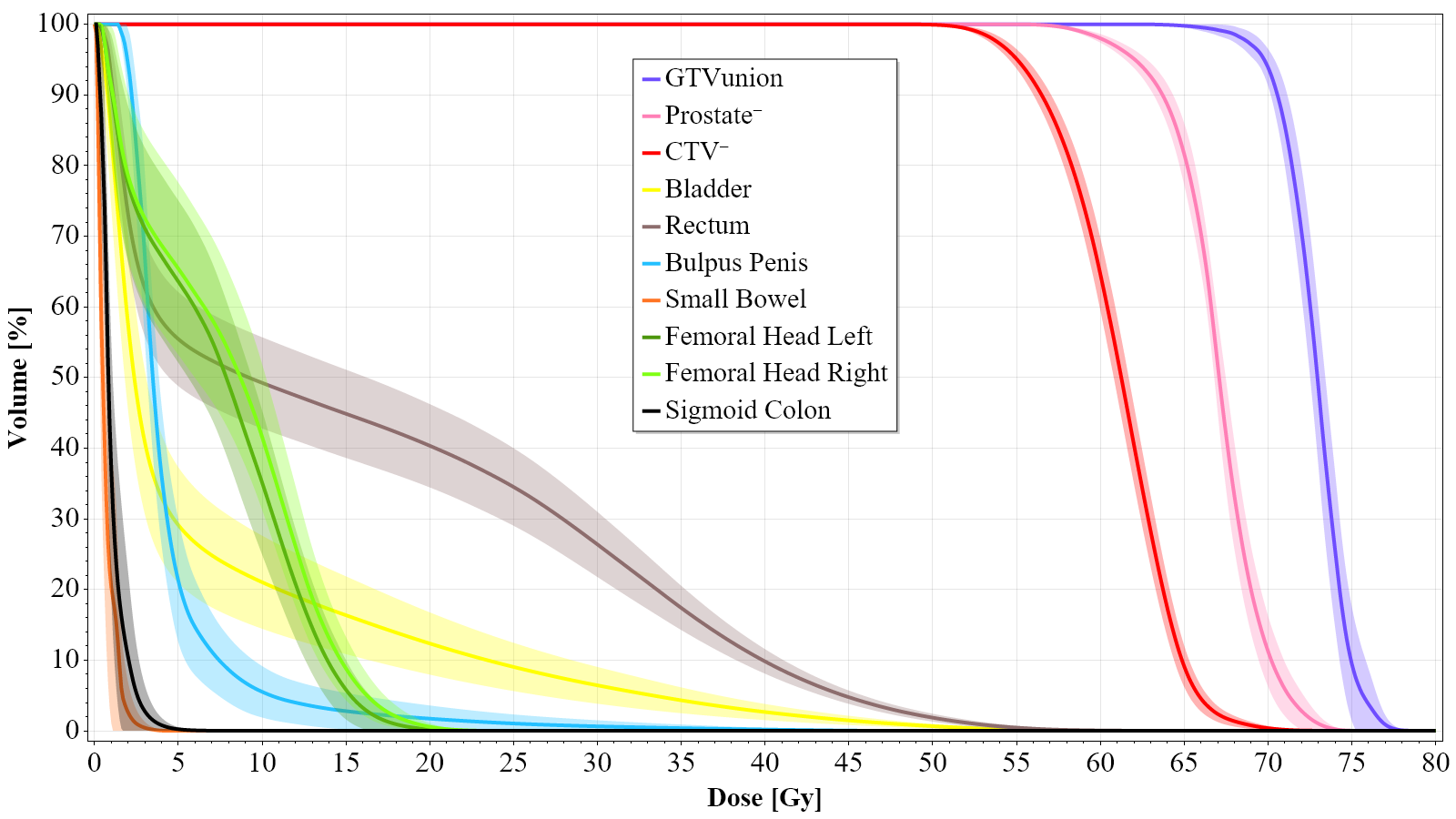


Figure S7: DVHs of the highest P_+_ plans for all 17 cases. The bold solid line represents the mean DVH, and the shaded area indicates the 95% confidence interval. Dose is the total physical dose delivered in 20 fractions.

# References

1. Groen VH, Haustermans K, Pos FJ, et al. Patterns of Failure Following External Beam Radiotherapy With or Without an Additional Focal Boost in the Randomized Controlled FLAME Trial for Localized Prostate Cancer. *European Urology*. 2022;82(3):252-257. doi:10.1016/j.eururo.2021.12.012

2. Spohn SKB, Sachpazidis I, Wiehle R, et al. Influence of Urethra Sparing on Tumor Control Probability and Normal Tissue Complication Probability in Focal Dose Escalated Hypofractionated Radiotherapy: A Planning Study Based on Histopathology Reference. *Front Oncol*. 2021;11. doi:10.3389/fonc.2021.652678

3. Künzel LA, Leibfarth S, Dohm OS, Müller AC, Zips D, Thorwarth D. Automatic VMAT planning for post-operative prostate cancer cases using particle swarm optimization: A proof of concept study. *Physica Medica*. 2020;69:101-109. doi:10.1016/j.ejmp.2019.12.007

4. Miften M, Olch A, Mihailidis D, et al. Tolerance limits and methodologies for IMRT measurement-based verification QA: Recommendations of AAPM Task Group No. 218. *Med Phys*. 2018;45(4):e53-e83. doi:10.1002/mp.12810
